# Spatiotemporal variations in gene expression, histology and biomechanics in an ovine model of tendinopathy

**DOI:** 10.1371/journal.pone.0185282

**Published:** 2017-10-12

**Authors:** Sara Biasutti, Andrew Dart, Margaret Smith, Carina Blaker, Elizabeth Clarke, Leo Jeffcott, Christopher Little

**Affiliations:** 1 Research and Clinical Training Unit, University Veterinary Teaching Hospital, University of Sydney, Camden, Australia; 2 Raymond Purves Bone and Joint Research Laboratory, The Kolling Institute, Sydney Medical School, The University of Sydney, Sydney, Australia; 3 The Institute of Bone and Joint Research, Royal North Shore Hospital, Sydney, Australia; 4 Murray Maxwell Biomechanics Laboratory, The Kolling Institute, Sydney Medical School, The University of Sydney, Sydney, Australia; University of Crete, GREECE

## Abstract

Flexor tendinopathy is a common problem affecting humans and animals. Tendon healing is poorly understood and the outcomes of conservative and surgical management are often suboptimal. While often considered a localized injury, recent evidence indicates that in the short term, tendinopathic changes are distributed widely throughout the tendon, remote from the lesion itself. Whether these changes persist throughout healing is unknown. The aim of this study was to document gene expression, histopathological and biomechanical changes that occur throughout the superficial digital flexor tendon (SDFT) up to 16 weeks post-injury, using an ovine surgical model of tendinopathy. Partial tendon transection was associated with decreased gene expression for aggrecan, decorin, fibromodulin, tissue inhibitors of metalloproteinases (*TIMPS 1*, *2* and *3*), collagen I and collagen II. Gene expression for collagen III, lumican and matrix metalloproteinase 13 (*MMP13*) increased locally around the lesion site. Expression of collagen III and *MMP13* decreased with time, but compared to controls, collagen III, *MMP13* and lumican expression remained regionally high throughout the study. An increase in *TIMP3* was observed over time. Histologically, operated tendons had higher pathology scores than controls, especially around the injured region. A chondroid phenotype was observed with increased cellular rounding and marked proteoglycan accumulation which only partially improved with time. Biomechanically, partial tendon transection resulted in a localized decrease in elastic modulus (in compression) but only at 8 weeks postoperatively. This study improves our understanding of tendon healing, demonstrating an early ‘peak’ in pathology characterized by altered gene expression and notable histopathological changes. Many of these pathological changes become more localized to the region of injury during healing. Collagen III and *MMP13* expression levels remained high close to the lesion throughout the study and may reflect the production of tendon tissue with suboptimal biomechanical properties. Further studies evaluating the long-term response of tendon to injury (6–12 months) are warranted to provide additional information on tendon healing and provide further understanding of the mechanisms underlying the pathology observed in this study.

## Introduction

Tendon injury and tendinopathies are common in both human and veterinary medicine. In humans, an increased participation in sport is associated with a higher incidence of tendon injury [1–4]. However, Achilles tendinopathy does not exclusively occur in human athletes [5–7], it is also reported to occur in the general population [8–10]. In equine athletes that train or race on the flat, prevalence rates of forelimb superficial digital flexor (SDF) tendinitis have been reported to be 11.1% [11], while the prevalence of ultrasonographic evidence of pathology in the forelimb SDF tendon (SDFT) in National Hunt horses, which are required to jump obstacles while racing, was found to be 24% [12]. In both humans and horses, outcomes following a diagnosis of tendinopathy are highly variable, but often suboptimal, with significant rates of re-injury and/or injury in the contralateral limb [13–17].

The pathophysiology of tendinopathy and tendon rupture remains unclear [18], however accumulated microtrauma, combined with an ineffective healing response, are currently thought to be the principal contributing factors [18, 19]. The mechanisms underlying the increased risk of reinjury or injury of the contralateral limb are also not well understood. Consequently, developing a better understanding of tendon healing may open the door to novel therapeutic strategies for this condition. Pathological tendon tissue arising from naturally occurring tendinopathy displays many similar features to experimentally injured tendons. Histopathological changes commonly seen in both pathological and healing tendon include proteoglycan accumulation, collagen fibre disorganization, increased blood vessel infiltration, increased cellularity and cellular rounding [20–22]. A chondroid cell phenotype, with increased glycosaminoglycan content, as well as increased cellularity, and cellular rounding has also been reported in both pathologic and healing tendons [21–24]. In addition to histological changes, many clinical and experimental studies have shown widespread alterations in gene expression in pathological and healing tendons. Common findings include increased expression of proteoglycans, variable changes in expression of fibrillar collagens, and disturbances in expression of matrix metalloproteinases (MMPs) and tissue inhibitors of MMPs (TIMPs) at the site of the lesion [25–30]. Recent studies at early post-injury time-points suggest that changes in tendon structure are not localized solely to the area of injury, but distributed up and down the tendon [22, 31]. These widespread changes have been suggested to contribute to a risk of re-injury at a site away from the initial lesion [22], previously observed in equine studies [23]. It is unknown whether these changes in areas remote from the lesion persist over time. Additionally, changes in tendon structure after injury may not only be restricted to the affected limb; it is recognized that unilateral tendon injury creates changes in the contralateral limb, and injury to the contralateral tendon is a common occurrence after Achilles tendon rupture [17, 32, 33]. The mechanisms underlying these changes are poorly understood. Currently, there are few models available for regional and temporal studies of injured tendon or their contralateral counterparts because of the inherently traumatic nature of sample collection and the subsequent pathology that may develop at the harvest site. Small biopsies provide valuable information about the intratendinous environment but are limited in many respects for evaluating the healing process because the early stages of tendinopathy are often asymptomatic, the initiation of tendon healing and reconstitution is not easily identified, and small samples are not generally representative of the entire tendon. The authors have developed a number of surgical animal models of tendinopathy where tendons can be sampled regionally and/or longitudinally in order to study the histopathologic, gene expression and biomechanical changes that occur throughout the tendon and whether these changes resolve over time.

The aim of this study was to identify the spatiotemporal changes in gene expression, histopathology and biomechanics that occur throughout injured tendons, using an ovine model of surgically-induced tendinopathy. As tendinopathic changes observed in previous surgical models have been widespread, away from the site of the lesion, the focus of this study was the regions adjacent and remote to the injury itself. We also hypothesized that any chondroid changes in the transected tendons would improve or resolve with time.

## Materials and methods

### Ethics statement

All procedures were approved by the University of Sydney’s Animal Ethics Committee (AEC no. N00/7-2013/3/6028).

### Animal allocation and treatment

Twenty-one, healthy, two year-old merino wethers were used in this study. Sheep were maintained under grazing conditions supplemented with lucerne hay as required. A sample size of six had been previously shown to be sufficient to detect a two-fold change in gene expression with 80–98% power depending on the gene [26]. Sheep were randomly allocated to three groups (7 sheep in each group). Eighteen sheep (6 in each group) underwent a 50% transection of a hindlimb superficial digital flexor tendon (SDFT) under general anesthesia (“transected”). Left or right limb distribution was randomly allocated. One sheep in each group underwent a sham operation (“sham operated control”) where the tendon was approached surgically but no transection was performed. The contralateral SDFT was used as a non-operated control tendon in all sheep (“non-operated control”).

#### Tendon surgery

Sheep were pre-medicated with diazepam (0.2mg/kg IV) prior to placing an intravenous catheter into the jugular vein. A combination of diazepam (0.1mg/kg IV) and ketamine hydrochloride (5mg/kg IV) was used for anesthetic induction, a cuffed, endotracheal tube was passed, and anesthesia was maintained using isoflurane in oxygen. Ceftiofur sodium (2.2mg/kg IV BID) was administered for 24 hours. Methadone (0.1mg/kg IV) and flunixin meglumine (1.1mg/kg IV) were administered for analgesia prior to surgery and a further dose of flunixin meglumine was given 12 hours later.

**Fig 1:**
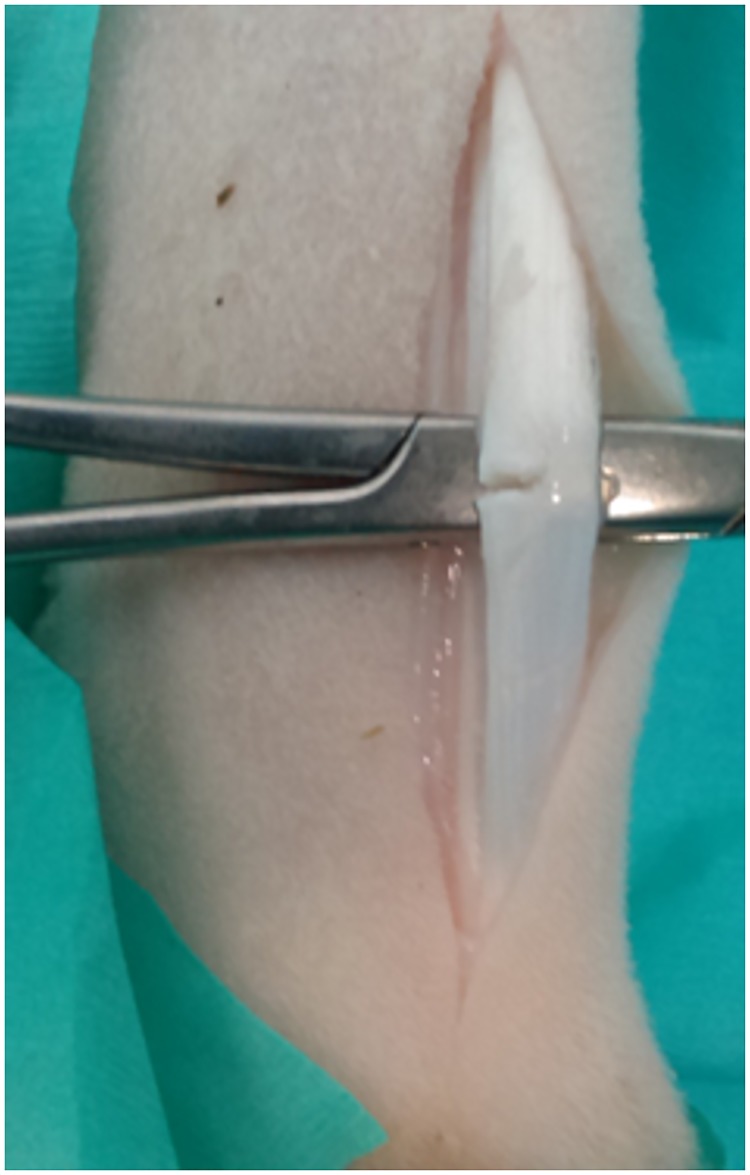
Surgical technique. The superficial digital flexor tendon (SDFT) was isolated and elevated prior to a 50% hemitransection on the lateral side using a no. 11 scalpel blade.

Following aseptic preparation of the surgical site, a 5cm incision was made over the plantarolateral aspect of the flexor tendons at the midpoint of the metatarsus. The SDFT was isolated, elevated with hemostats, and the lateral 50% of the tendon was transected using a scalpel (Fig 1). In sham operated subjects the tendon was elevated but not transected. The subcutaneous tissue and skin were closed with absorbable sutures and a bandage applied to the limb. The sheep were housed in groups, in stalls, for 7 days. There were no postoperative complications and the sheep were fully weight-bearing the day following surgery. After 7 days, bandages were removed, and the sheep were returned to pasture where they were monitored daily. Sheep were euthanased at 8, 12 and 16 weeks postoperatively using an overdose of sodium pentobarbitone intravenously.

#### Sample collection

**Fig 2:**
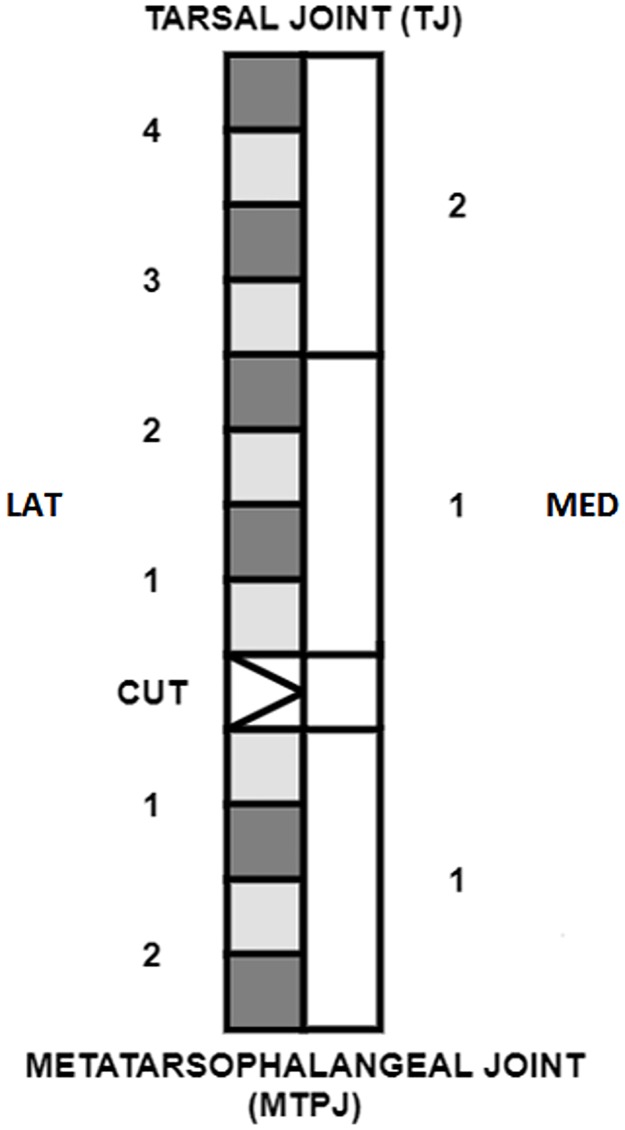
Sample collection schematic. “LAT” and “MED” indicate the lateral and medial sides of the tendon and the numbers indicate the regional division of the tendon. On the lateral side, the light grey regions were sampled for histopathology and the dark grey regions for gene expression analysis. On the medial side, the white regions were sampled for changes in compressive modulus.

Immediately following euthanasia the hindlegs of all sheep were shaved and cleaned with 70% isopropyl alcohol. An incision was made longitudinally along the plantar aspect of the hindlimb from the tip of the calcaneus proximally to the metatarsophalangeal joint distally. The SDFT was exposed, isolated and transected at its most proximal and most distal ends. The tendon was wrapped in a gauze swab moistened with sterile saline, placed into a sealed bag, on ice and processed within 8 hours of euthanasia. To obtain samples, the paratenon was removed and the tendon was halved longitudinally. The medial side was processed for biomechanical assessment and the lateral side for histopathology and gene expression analysis (Fig 2). Due to the greater proximal length of the SDFT (which extends proximal to the metatarsus to insert on the calcaneus), 4 regions were available proximal to the lesion, while only 1–2 regions could be harvested distal to the lesion (Fig 2). The lesion created by the hemitransection (approximately 1cm in length) in the transected tendons was removed from the lateral side and the corresponding tissue from the medial half of the tendon was also removed. This tissue was not analyzed as the focus of this study was the regions adjacent to and remote from the injury rather than the lesion itself. In sham operated and non-operated control tendons, the corresponding region from the mid-body of the tendon was removed to create sections of equal length between the operated and control tendons. Each side of the tendon was then divided regionally. The lateral side was divided into 2.5cm sections which were split transversely into 1.25cm sections for histology and gene expression analysis. Four regions were collected from the proximal section and up to two sections distal to the lesion, depending on tendon length. The medial side was divided into 5cm sections for biomechanical testing. Each section was marked with 5% Alcian blue dye at the proximal end, opposite the longitudinal central transection, to orientate the samples for histopathology and biomechanical testing.

Histology samples were placed directly in 10% (v/v) neutral buffered formalin without removal of any further surrounding tissue. Samples for gene expression analysis were trimmed of any residual soft tissue or epitenon and snap-frozen in liquid nitrogen before being stored at -80°C. Samples for biomechanical testing were wrapped in sterile saline-soaked gauze swabs before being stored in 4ml sample tubes and frozen at -20°C.

#### Gene expression analysis

Processes for RNA extraction, real time polymerase chain reaction (qPCR) and complementary DNA (cDNA) analysis were performed in our laboratories within weeks of sample collection as previously described [22]. Briefly, samples for gene expression analysis were weighed and trimmed over dry ice (to between 80 – 150mg) before being powdered in a Dismembrator (Braun, Melsungen, Germany). Extraction was accomplished with TRIzol reagent (Invitrogen, Melbourne, VIC, Australia) chloroform and RNeasy MiniKits (Qiagen, Doncaster, VIC, Australia) with an on-column DNase step (RNase-Free DNase Set; Qiagen) to eliminate any genomic DNA contamination as per manufacturer recommendations. Quantification of RNA concentration and sample quality assessment was performed using a Nanodrop spectrophotometer (Thermo-Fisher Scientific, Australia). RNA purity was confirmed prior to qPCR using a no-reverse transcription (no-RT) qPCR step to check for any contaminant genomic DNA. One microgram of RNA from each sample was reverse transcribed in a reaction volume of 50μL for 3 hours at 37°C using RT kits (GoScript from Promega) as per manufacturer instructions except random pentadecamers (Sigma Genosys, North Ryde, NSW, Australia) instead of hexamers were used. cDNA was stored at 4°C until analysed by real time qPCR using Immomix (Bioline), and SYBRGreen II (Cambrex) in a Rotorgene 6000 analyser (Corbett Life Sciences, Mortlake NSW, Australia). Eighteen genes were analysed and specific primers (Sigma Genosys, North Ryde, NSW, Australia) were as previously published (S1 Table) [26]. The RotorGene real-time qPCR software program was used to determine the threshold cycle (Ct, the cycle at which the fluorescence in the tube increases significantly above background). For each cDNA sample, the threshold cycle was converted to a relative fluorescence unit (RFU) by interpolation of a standard curve as validated in previous literature [34]. This method (automated by the Rotorgene software) gives similar relative values to both the delta–delta Ct and the Pfaffl methods [34] and has advantages by detecting efficiency and high cycle inhibition. Normalisation against a control gene was not performed as the usual reference genes present at the same concentration of the genes of interest change significantly with alteration of load on musculoskeletal tissues [35]. The fold difference of expression was then calculated by dividing each sample RFU by the baseline non-operated control value of each gene for the entire tendon. Melt curves were generated after each qPCR run to confirm a single identical gene-specific amplification product.

#### Histology

Histology samples were processed as previously described [22]. Briefly, samples were fixed in 10% (v/v) neutral buffered formalin for 48 hours then dehydrated in ethanol (v/v) for 36 hours. The specimens were then cleared in methyl benzoate for 72 hours prior to infiltration with 1% and 5% (w/v) celloidin (both with 1.5% (w/v) tricresyl phosphate in methyl benzoate) for 72 hours and 3 weeks, respectively. Samples were rinsed with chloroform and infiltrated with paraffin wax before being embedded in paraffin blocks. Prior to cutting, the blocks were softened with 5% formic acid, 45% ethanol and 50% glycerol on a cold plate and rinsed in cold water. Five micron sections were then cut using a rotary microtome (Leica RM2255, Leica Microsystems, Wetzlar, Germany). Sections were stained with haematoxylin and eosin (H & E), picrosirius red (PSR) or toluidine blue.

Histology slides were scored by three blinded observers (MS, SB and NM) according to a previously described grading system (S2 Table) [22, 26]. Slides stained with H & E were scored for the following: intrafascicular cellularity, intrasfascicular tenocyte morphology, interfascicular inflammatory cell infiltration, and vascularity. Intrafascicular and interfascicular cells were distinguished on the basis of their position within, or external to, the visible tendon fascicle. Inflammatory cells were characterised based on their cell morphology. Vascularity was assessed on the basis of the number as well as the size of vessels observed. PSR slides were scored for collagen fibre alignment (under optimised polarised light) and toluidine blue slides for proteoglycan accumulation. Where a semi-subjective assessment was required (such as mild/moderate/marked for cellularity, inflammatory cell infiltration, vascularity and proteoglycan content) observers were shown representative images for reference from previous studies [22, 26] prior to commencing scoring. Histological parameters were based on features of the tendon substance; epitenon was not included in the scoring. All categories were scored 0–3, where 0 equates to a normal tendon and 3 indicates a highly pathological tendon (S2 Table). A total histopathology score (0–15) was then calculated when all scores (except for proteoglycan score) were added; proteoglycan score was excluded as a component of the total histopathology score as an increase in proteoglycan accumulation is a normal finding in the insertional regions of tendons and may falsely elevate the total histopathology score. A higher score indicated a tendon which was more pathological in appearance. Images of sections were obtained using a light microscope and digital camera with Image Manager software (Leica Microsystems).

#### Immunohistochemistry

Sections from the lateral half of the tendon directly proximal and distal to the lesion or midmetatarsus which demonstrated the median gene expression level for collagen III, MMP13 and lumican were prepared. For collagen III, sections were pretreated with proteinase K (1:10 solution, #S3020, Dako) for 30 minutes at room temperature prior to pretreatment with bovine testicular hyaluronidase (Sigma #H3505-5G, 1000U/ml for 2 hours at 37°C). Slides were rinsed then incubated with affinity-purified rabbit polyclonal antibodies to full-length collagen III aa 1–1466 (Abcam ab7778, 1.25ug/ml) overnight at 4°C. For MMP13, samples were pretreated with bovine testicular hyaluronidase (Sigma #H3505-5G, 500U/ml) for 30 minutes at 37 C. Slides were washed and incubated with primary antibodies to rabbit MMP13 (LS-B3168 Lifespan Polysciences, 0.83ug/ml) overnight at 4°C. For lumican, samples were pretreated as for MMP13 prior to rinsing and incubation with an anti-lumican antibody (provided by Dr Emily Fuller, Raymond Purves Laboratory, the Kolling Institute at the University of Sydney, 1.0ug/ml) overnight at 4°C. Antibody binding was detected with rabbit EnVision (#K4003 for collagen III, Dako) and mouse EnVision (#K4001 for MMP13 and lumican, Dako) for 30 minutes at room temperature, then ImmPACT NovaRED (#SK 4805 for all samples, Vector) for 15 minutes at room temperature prior to counterstaining with Mayer’s haematoxylin for 5 minutes. Equivalent concentrations of species-matched immunoglobulins on identically treated sections were used as a negative control.

Immunohistochemistry slides were scored by one observer (SB). Each section was scored from 1–3, as follows: 1 = mild staining, 2 = moderate staining, 3 = marked staining. The distribution of staining was also noted as either pericellular or within the matrix.

#### Biomechanics

In order to evaluate whether injured tendon becomes biomechanically chondroid in nature, samples were tested in compression rather than in tension. Samples were thawed and trimmed to a cuboid shape to enable testing in compression. After trimming, each sample was measured in three dimensions using Vernier calipers. Each dimension was measured three times and averaged. The cross-sectional dimensions were used to calculate tendon cross sectional area for normalisation of force to stress, and the height was used for normalisation of displacement to strain. The tendon was then placed on a platform with the tendon fibres orientated parallel to the platform. A flat platen was lowered onto the specimen until a threshold force of 0.05 N was reached. The specimen was tested in unconfined compression (without preconditioning) to 35% strain at a strain rate of 10%/s. Force (2.5 lb load cell, MDB-2.5, Transducer Techniques, Temecula, CA) and displacement were measured throughout the test at 1615 Hz. Force and displacement were converted to stress and strain, and the elastic modulus was measured as the average gradient of the stress-strain curve at three different strains (10, 15 and 20%), over a range of +/-1% strain.

#### Statistical analysis and data visualization

Statistical analysis was performed using Stata version 14. Raw gene expression data was not normally distributed so results were normalized using a logarithmic transformation prior to analysis. There were no significant differences between sham operated and non-operated control tendons so these were pooled (‘control tendons’) for multivariate analysis. Multivariate analysis using mixed regression modelling of each gene was carried out in the first instance with data clustered by sheep and limb. Where differences were identified in the mixed model, univariate analysis was carried out to determine differences between groups (either transected or control tendons) or within groups (for transected and control tendons at each different time point). As transected and control samples arose from the same animal, gene expression differences between transected and contralateral control tendons at each time point were determined using a paired t-test on normalized data. A one way analysis of variance (ANOVA) was then used to test the control tendons for differences between time points. The fold change between transected and control tendons in each region was calculated for each gene using the equation: RFU of each region/mean RFU control tendons. This fold change data was not normally distributed, so differences between time points within different regions were calculated using a Kruskal-Wallis analysis, followed by a Mann-Whitney U test (for unpaired data) where differences between time points were observed. As absolute values of *TIMP* and *MMP* expression vary between individuals, it has been suggested that relative expression of *TIMP*s and *MMP*s may be more relevant in assessing a patient for ongoing collagenolysis [36, 37]. To determine a relative ratio of possible activity and inhibition of MMPs, the ratio of *TIMP1*:*MMP13* was calculated for all samples as previously described [37]. The ratio was normalized with a log transformation, then analysed with mixed regression modelling and differences between transected and control tendons analysed using a Mann-Whitney U test. Differences between time points for normalized ratio data were assessed using a one-way ANOVA.

Histopathology data were ordinal, so multivariate analysis was performed using mixed ordinal logistic regression. A Benjamini-Hochberg post-hoc correction was applied. Significance was determined to be *P* < 0.025 for these regression results after correction. Differences between cut and control tendons at each time point were analyzed using a Wilcoxon signed rank test to account for pairing of data. Differences in transected tendons between time points were analysed using a Kruskal-Wallis analysis followed by a Mann-Whitney U test (for unpaired data).

Partial correlation coefficients were generated as previously described using Kendall’s tau-b analysis to determine associations between gene expression and histopathology [22], with the subprogram, PARTTAU (James Fiedler and Alan Feiveson, Johnson Space Centre, Houston, Tx, USA), used within Stata to correct for confounders (surgery and region of original sample). For each gene association within each group (control or transected, and transected tendons at 8, 12 and 16 weeks), the Benjamini-Hochberg correction was applied to the relevant P value.

Biomechanics data were analysed using mixed regression modelling to determine multivariate effects, then differences between transected and control tendons at each time point were analysed using the Wilcoxon signed rank test. Differences between time points were determined with a Kruskal-Wallis analysis followed by a Mann-Whitney U test.

Results are presented graphically as box plots. The median is marked and hinges are present at the 25^th^ and 75^th^ percentile. The graphs are presented with the tendon region oriented horizontally with proximal to the left and distal to the right of the graph. “CUT” denotes the position of the lesion which was removed and not analysed in this study. Gene expression results are presented on a logarithmic scale as relative fluorescent units (“RFU”) per microgram of RNA; results were not normalized to GAPDH in this study as GAPDH was noted to vary over time in control tendons.

## Results

### Gross pathology

**Fig 3:**
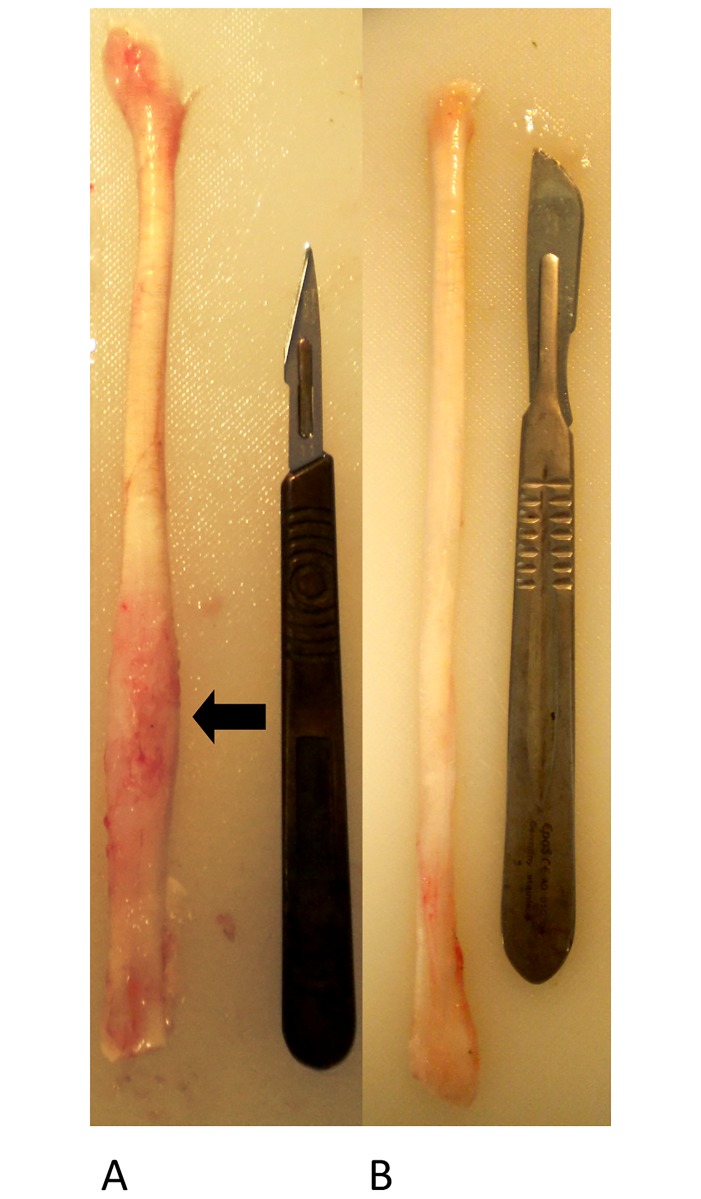
Representative sample of transected tendon (A) and control tendon (B). In comparison to control tendon, the transected tendon is visibly thickened and pink in colour distally in the regions surrounding the transection. The area of the lesion is poorly demarcated (arrow).

At all time points, tendons were easily removed from their surrounding sheaths and no visible established adhesions were noted between the paratenon and tendon sheath. The interior of the tendon sheath appeared normal and no adhesions or evidence of synovitis were noted in this region. The distal half of the tendon was visibly thickened and pink in colour, in contrast to the usual white appearance of normal tendon and the more proximal regions of the transected tendons (Fig 3). The paratenon appeared to be focally proliferative in the region of the transection, with multiple fine pinkish strands visible on the tendon surface. The epitenon of control tendons was easily removed for gene expression samples, but in transected tendons it was firmly adhered to the region of the transection and required sharp dissection to remove. Control tendons were off-white in colour, firm and resilient in texture and were difficult to cut transversely with a scalpel blade, while transected tendons (in the regions close to the transection) were much more easily cut. Subjectively, the tendons harvested at the later time points (12 and 16 weeks) appeared slightly less thickened and were a paler pink in colour at the lesion site than tendons harvested at 8 weeks, but no other gross differences were appreciated.

### Histology

#### Control tendons

Normal (control) tendons showed histologic heterogeneity both with regard to region and time (S3 Table). The cellularity (OR 0.21, *P* < 0.001), cell morphology (OR 0.19, *P* < 0.001), vascularity (OR 0.35, *P* < 0.001), cell infiltration (OR 0.20, *P* < 0.001), sum of scores (OR 0.23, *P* < 0.001), and proteoglycan content (OR 0.24, *P* < 0.001) were highest in the mid tendon (at the site where the lesion is made in transected tendons), with the likelihood of a higher score decreasing with distance from this site. Proteoglycan score was also likely to be higher in the tendon distal to the region equivalent to the lesion site in transected tendons compared to the proximal tendon (OR 4.8, *P* = 0.001). There was an effect of time on cell morphology, where the risk of a higher score decreased over time, reflecting a progressive decrease in rounded cells (OR 0.6, *P* = 0.009). When the effect of time was analysed alone, the cell morphology score decreased in the region proximal to the mid-tendon (*P* = 0.048) between 8 weeks and 12 weeks (*P* = 0.026).

#### Transected tendons

**Fig 4:**
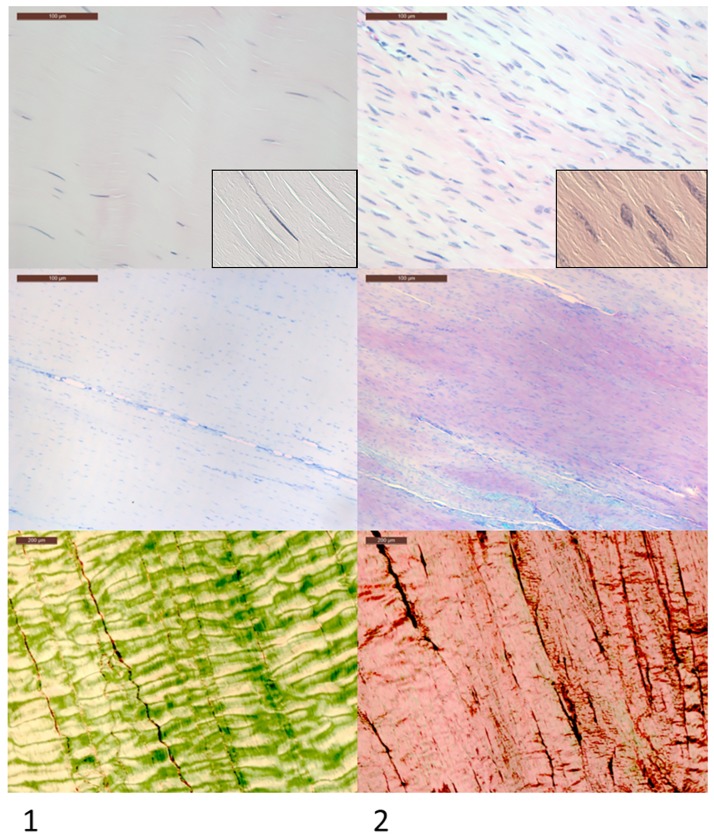
Histological appearance of normal and healing tendon. Column 1 shows the typical microscopic appearance of normal tendon in contrast with healing transected tendon in Column 2. Magnified cell morphology images are displayed in inset panels. Contralateral uninjured tendons were used as controls as well as a single sham operated subject in each group. Transected tendon has markedly increased cellularity with a more rounded cell morphology (seen in inset panels), increased proteoglycan accumulation (purple staining) and collagen fibre malalignment under polarized light. The cellular rounding and increased proteoglycan staining is indicative of a chondroid phenotype.

Tendon transection increased the risk of a higher overall histopathology score (OR 17 (confidence interval 8–38), *P* < 0.001) and proteoglycan accumulation (OR 2.6 (1.3–5.3), *P* < 0.007) (Fig 4, S3 Table). There was a higher risk of an increased proteoglycan in the tendon distal to the lesion site compared to the proximal tendon (OR 2.2 (1.2–4.2), *P* = 0.015). Transection increased the odds of a higher score for all the following individual pathology parameters; cellularity (OR 14 (7–28), *P* < 0.001), cell morphology (OR 12 (6–23), *P* < 0.001), vascularity (OR 11 (6–23), *P* < 0.001), interfascicular cell infiltration (OR 58 (17–196), *P* < 0.001) and loss of collagen fibre alignment (OR 10 (5–21), *P* < 0.001) (Fig 4). Overall, for all variables, pathology scores were higher close to the lesion, with the odds of a higher pathology score decreasing as distance from the lesion site increased (OR <0.5, *P* < 0.001 for all variables) (S3 Table).

**Fig 5:**
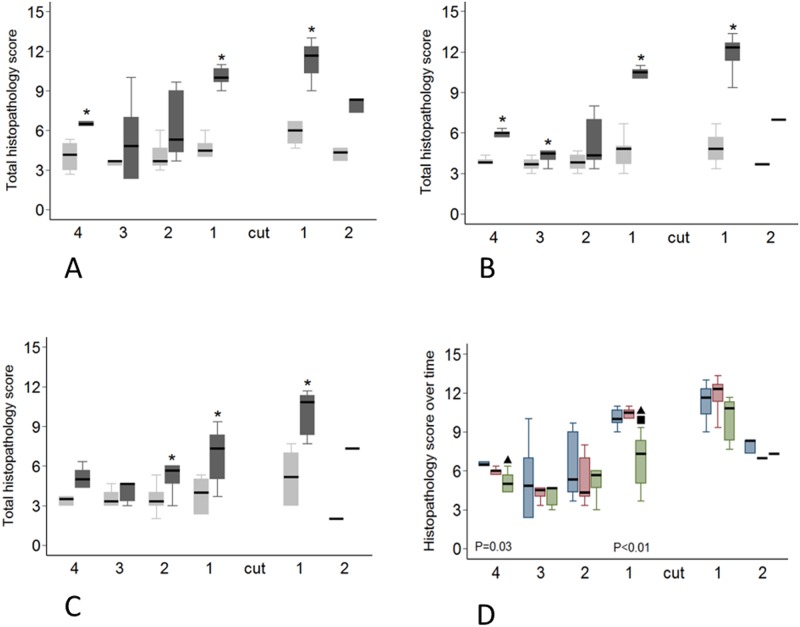
Regional variation in histopathology score. Transected tendons are represented by the dark grey box and control tendons by the light grey box. Significant differences (P<0.05) between transected and control tendons as determined with a Wilcoxon signed-rank test are indicated by the asterisk. A-C: Variation in histopathology score at 8 weeks (A), 12 weeks (B) and 16 weeks (C). D: Histopathological score in transected tendons at 8 (blue boxes), 12 (red boxes) and 16 weeks (green boxes). Significant differences between time points (P<0.05, determined with a Mann-Whitney U test) are indicated by the black triangles (8 weeks significantly different to 16 weeks) and the black square (12 weeks significantly different to 16 weeks).

There was a regional and temporal variation in the histopathology in transected compared to control tendons (Fig 5A–5D). In transected tendons the sum of scores (OR 0.85 (0.74–0.97), *P* = 0.017), cell morphology score (OR 0.83 (0.72–0.96), *P* = 0.012) and inflammatory cell infiltration score (OR 0.80 (0.69–0.94), *P* = 0.005) decreased over time. When the effect of time was analyzed alone, a localized response was noted, the total histopathology score decreasing in the most proximal region (*P* = 0.034) and in the region directly proximal to the transection (*P* = 0.0057). In the most proximal tendon region this reduction was observed between 8 weeks and 16 weeks (*P* = 0.035) while in the region directly proximal to the transection the total score decreased after 12 weeks (*P* = 0.0064) (Fig 5D). Cell morphology score decreased in the region directly proximal to the transection (*P* = 0.0098) and this reduction was noted after 12 weeks. There was no difference between the 8 and 12 week score but the score at 16 weeks was lower than at both 8 weeks (*P* = 0.016) and 12 weeks (*P* = 0.0057). The inflammatory cell infiltration score also decreased in the most proximal region (*P* = 0.035) and in the region directly proximal to the transection (P = 0.0063). This decrease occurred after 12 weeks in both regions (*P* = 0.0051 and *P* = 0.022 respectively). Based on the results of the mixed ordinal logistic regression analysis cellularity, vascularity, collagen fibre alignment and proteoglycan scores did not change with time in transected tendons (S3 Table).

### Biomechanics

**Fig 6:**
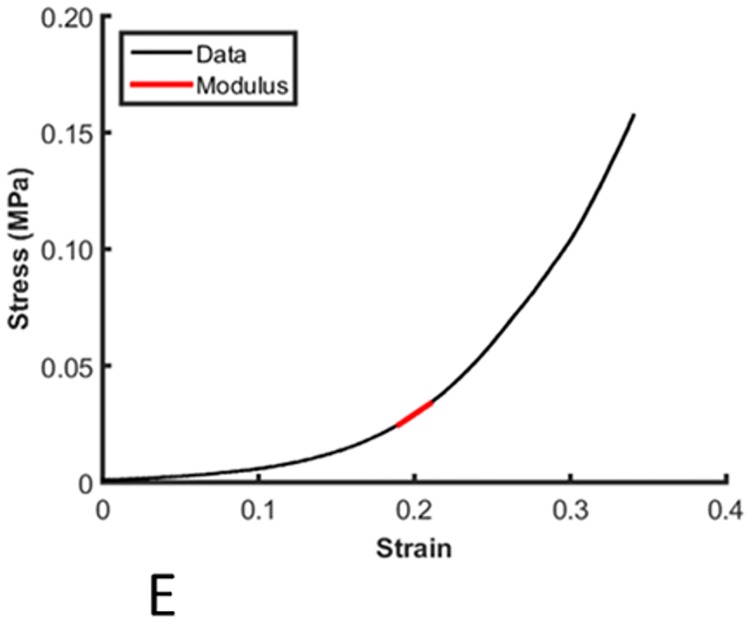
Modulus measurement on the stress-strain curve. A typical stress strain curve from a compression test at 20% strain, with the modulus measurement point indicated by the red line.

**Fig 7:**
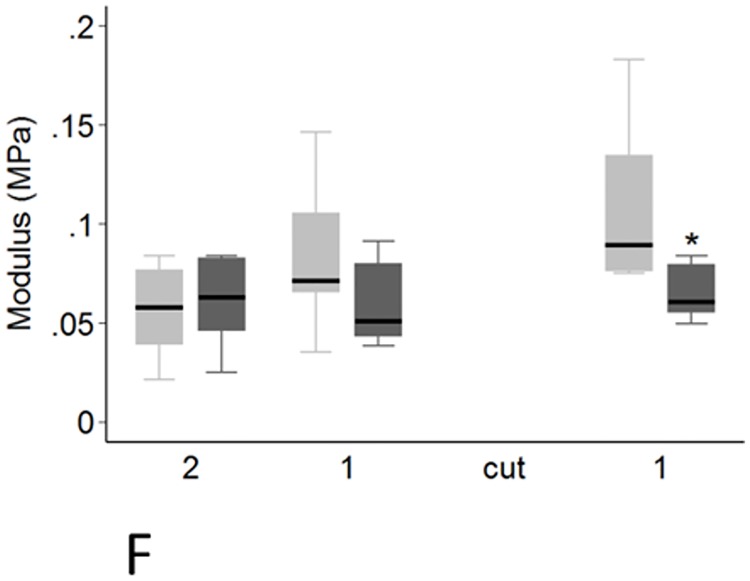
Variation in compressive elastic modulus at 10% strain, 8 weeks post-transection. The tendon is orientated horizontally with proximal to the left and distal to the right with regional allocation as per Fig 2. A significant reduction in modulus in transected tendons (dark grey boxes) was observed in the region distal to the transection (asterisk).

The compressive elastic modulus of control and transected tendons was calculated routinely (Fig 6). The average elastic moduli of transected tendons measured in unconfined compression at 20, 15 and 10% strain were 0.28 ± 0.16 MPa, 0.15 ± 0.12 MPa and 0.07 ± 0.03 MPa, respectively. Multivariate analysis showed no differences in compressive modulus at 20% or 15% strain between transected and control tendons, but there was a significant decrease in modulus in transected tendons at 10% strain (*P* = 0.029). The differences between transected and control tendons were only identified at 8 weeks post-injury in regons adjacent to the lesion, particularly distal to the injuy (*P* = 0.04 in pairwise analyses; Fig 7).

### Gene expression

We investigated the potential underlying molecular mechanisms driving the spatio-temporal pathology in transected tendons by comparing the expression of key structural proteins and matrix remodelling enzymes and their inhibitors. Mean RNA yields for the different experimental groups at 8, 12 and 16 weeks were 63.40, 48.60, and 84.12 ng/mg for control tendons, and 85.08, 63.25, and 69.04 ng/mg for transected tendons. A summary for the results of the mixed regression analysis for gene expression is presented in Tables 1 and 2. Effects of time after surgery and tendon region (proximal location (close to the tarsus) vs distal location (close to the metatarso-phalangeal (MTP) joint), or distance from lesion site (either proximal or distal)) on gene expression were evaluated separately for control tendons, then for all (control plus transected) tendons. For control tendons, models for *ACAN*, *VCAN*, *DCN*, *FMOD*, *LUM*, *COL2A1*, *ADAMTS4*, *MMP2*, and *TIMP1* were not significant (P > 0.05 for the overall statistical model), but for *ACAN*, *COL2A1*, *ADAMTS4*, *and MMP2* there were significant differences for some covariates (*P* < 0.035 after Benjamini-Hochberg correction for false positives). For transected tendons, all models aside from those for *ADAMTS4*, and *MMP9* were significant (*P* < 0.015), demonstrating spatio-temporal regulation of expression of these genes as described below. The results for the paired t-test analysis (pairwise analysis for direct comparison between specific regions in transected and control tendons) are presented in Table 3.

**Table 1:** Effects of surgery and spatial position on matrix gene expression by mixed regression modelling of log-transformed expression data for each gene.

Gene	Model *P*	Effect of transection	*P*	Effect of time after surgery as change per week	*P*	Effect of proximity to tarsus (T) or metatarso-phalangeal joint (MTP)	*P*	Effect of distance (per cm) from midmetatarsus (lesion site)	*P*
*ACAN*	0.096			⬆ 1.15 (1.02–1.30)	0.022	1.00 (0.73–1.35)	0.98	0.94 (0.84–1.06)	0.35
	<0.001	⬇ 0.56 (0.44–0.70)	<0.001	1.13 (1.00–1.29)	0.056	1.04 (0.78–1.37)	0.80	1.02 (0.92–1.14)	0.71
*VCAN*	0.20			1.05 (0.98–1.13)	0.19	1.21 (0.91–1.62)	0.20	1.10 (0.98–1.23)	0.11
	0.003	1.20 (0.95–1.51)	0.12	1.02 (0.94–1.10)	0.64	**T > MTP** 1.5 (1.1–1.9)	0.007	0.94 (0.84–1.04)	0.23
*DCN*	0.57			1.02 (0.94–1.10)	0.60	0.87 (0.70–1.10)	0.24	0.95 (0.87–1.04)	0.29
	<0.001	⬇ 0.65 (0.54–0.78)	<0.001	1.02 (0.94–1.12)	0.62	0.99 (0.80–1.24)	0.96	1.08 (0.99–1.17)	0.066
*BGN*	<0.001			1.02 (0.96–1.09)	0.51	1.17 (0.90–1.51)	0.24	⬇ 0.79 (0.72–0.88)	<0.001
	<0.001	1.03 (0.85–1.24)	0.78	1.00 (0.93–1.07)	0.96	1.18 (0.95–1.47)	0.13	⬇ 0.75 (0.69–0.81)	<0.001
*FMOD*	0.28			0.99 (0.92–1.07)	0.82	0.83 (0.66–1.04)	0.099	0.93 (0.85–1.01)	0.094
	<0.001	⬇ 0.65 (0.53–0.78)	<0.001	0.98 (0.91–1.07)	0.66	0.92 (0.75–1.13)	0.41	1.04 (0.96–1.12)	0.32
*LUM*	0.19			0.91 (0.84–1.00)	0.048	1.18 (0.82–1.69)	0.37	1.04 (0.91–1.20)	0.55
	<0.001	⬆ 2.0 (1.5–2.7)	<0.001	0.91 (0.82–1.01)	0.090	**T > MTP** 1.5 (1.1–2.2)	0.017	⬇ 0.80 (0.70–0.91)	0.001
*COL1A1*	<0.001			0.94 (0.83–1.07)	0.33	**MTP > T** 0.61 (0.45–0.84)	0.002	1.06 (0.94–1.20)	0.36
	<0.001	⬇ 0.66 (0.50–0.88)	0.004	0.92 (0.81–1.04)	0.18	0.76 (0.57–1.00)	0.051	⬆ 1.14 (1.03–1.27)	0.014
*COL2A1*	0.096			1.05 (0.93–1.19)	0.41	**MTP > T** 0.65 (0.45–0.93)	0.019	0.95 (0.82–1.09)	0.43
	<0.001	⬇ 0.54 (0.39–0.74)	<0.001	1.05 (0.93–1.20)	0.42	0.74 (0.54–1.00)	0.050	1.02 (0.90–1.14)	0.80
*COL3A1*	0.011			⬇ 0.89 (0.81–0.98)	0.017	**T > MTP** 1.5 (1.1–2.1)	0.024	1.10 (0.96–1.27)	0.16
	<0.001	⬆ 2.4 (1.8–3.2)	<0.001	⬇ 0.86 (0.77–0.95)	0.005	**T > MTP** 1.7 (1.2–2.4)	0.003	⬇ 0.81 (0.71–0.93)	0.002

Data was clustered by sheep and limb. For each gene, the results of the analysis in control tendons are listed first followed by results in transected tendons. Expression fold differences (as mean anti-log of beta-coefficients followed by the 95% confidence interval in parentheses) are given for models without transection (n = 129 observations; controls only) and with transection (n = 219; controls and operated) if model was significant (*P*<0.05) or one or more covariates was significant (*P*< 0.035 after Benjamini-Hochberg correction for false positives). Arrows indicate direction of change (increase or decrease).

**Table 2:** Effects of surgery and spatial position on enzyme and inhibitor gene expression by mixed regression modelling of log-transformed expression data for each gene.

Gene	Model *P*	Effect of surgery	*P*	Effect of time after surgery as change per week	*P*	Effect of proximity to tarsus (T) or metarso-phalangeal joint (MTP)	*P*	Effect of distance (per cm) from midmetatarsus (lesion site)	*P*
*ADAMTS4*	0.10			0.98 (0.93–1.04)	0.54	**MTP > T** 0.76 (0.59–0.97)	0.031	1.06 (0.96–1.17)	0.22
	0.20	0.82 (0.64–1.06)	0.13	0.96 (0.90–1.03)	0.28	0.86 (0.69–1.08)	0.19	1.01 (0.93–1.10)	0.84
*ADAMTS5*	<0.001			0.98 (0.93–1.03)	0.44	1.15 (0.93–1.43)	0.20	⬇ 0.76 (0.70–0.83)	<0.001
	<0.001	0.94 (0.81–1.09)	0.40	0.97 (0.91–1.03)	0.27	1.16 (0.97–1.38)	0.11	⬇ 0.73 (0.68–0.78)	<0.001
*MMP2*	0.17			0.99 (0.85–1.15)	0.88	0.72 (0.44–1.17)	0.18	⬇ 0.81 (0.67–0.98)	0.028
	0.014	1.10 (0.76–1.61)	0.60	0.99 (0.86–1.14)	0.86	0.78 (0.53–1.16)	0.21	⬇ 0.76 (0.66–0.89)	0.001
*MMP9*	0.20			0.92 (0.83–1.03)	0.15	0.72 (0.49–1.07)	0.11	0.95 (0.82–1.09)	0.45
	0.31	1.04 (0.80–1.34)	0.79	0.93 (0.84–1.04)	0.22	0.88 (0.64–1.20)	0.42	0.90 (0.80–1.01)	0.076
*MMP13*	<0.001			0.89 (0.80–0.99)	0.039	**T > MTP** 3.8 (2.0–7.6)	<0.001	⬇ 0.75 (0.57–0.97)	0.031
	<0.001	⬆ 20.0 (9.2–43.6)	<0.001	⬇ 0.86 (0.76–0.98)	0.027	**T > MTP** 2.9 (1.5–5.8)	0.002	⬇ 0.39 (0.30–0.51)	<0.001
*TIMP1*	0.35			0.97 (0.84–1.11)	0.65	0.86 (0.56–1.32)	0.49	0.86 (0.73–1.02)	0.080
	0.003	⬇ 0.57 (0.43–0.75)	<0.001	0.99 (0.85–1.15)	0.88	1.07 (0.76–1.50)	0.69	0.97 (0.85–1.10)	0.66
*TIMP2*	<0.001			1.06 (0.93–1.26)	0.37	**MTP > T** 0.45 (0.26–0.76)	0.003	⬇ 0.67 (0.54–0.82)	<0.001
	<0.001	⬇ 0.38 (0.40–0.78)	<0.001	1.08 (0.93–1.26)	0.33	**MTP > T** 0.59 (0.37–0.94)	0.028	0.84 (0.70–1.01)	0.061
*TIMP3*	<0.001			⬆ 1.22 (1.11–1.34)	<0.001	0.81 (0.48–1.38)	0.44	0.92 (0.75–1.14)	0.45
	<0.001	⬇ 0.56 (0.40–0.78)	0.001	⬆ 1.22 (1.11–1.34)	<0.001	1.00 (0.67–1.50)	0.99	1.04 (0.89–1.21)	0.65
*GAPDH*	0.048			1.07 (1.00–1.14)	0.037	0.96 (0.79–1.18)	0.71	0.93 (0.86–1.01)	0.073
	<0.001	⬇ 0.73 (0.63–0.83)	<0.001	1.06 (0.99–1.14)	0.10	1.01 (0.86–1.20)	0.86	0.99 (0.93–1.05)	0.71

Data was clustered by sheep and limb. For each gene, the results of the analysis in control tendons are listed first followed by results in transected tendons. Expression fold differences (as mean anti-log of beta-coefficients followed by the 95% confidence interval in parentheses) are given for models without transection (n = 129 observations; controls only) and with transection (n = 219; controls and operated) if model was significant (*P*<0.05) or one or more covariates was significant (*P*< 0.035 after Benjamini-Hochberg correction for false positives). Arrows indicate direction of change (increase or decrease).

**Table 3:** Gene expression differences in transected (“T”) and control (“C”) tendons at 8, 12 and 16 weeks post-transection.

GENE	8 WEEKS (P-value)		12 WEEKS (P-value)		16 WEEKS (P-value)	
Collagen I (*COL1A1*)	No significant differences		Prox:	Region 2, C>T 2.79 fold (0.031)	Prox:	Region 4, C>T 2.03 fold (0.025)
						Region 1, C>T 3.02 fold (0.015)
Collagen III (*COL3A1*)	Prox:	Region 1, T>C 16.72 fold (0.0075)	Prox:	Region 1, T>C 5.03 fold (0.0088)	Prox:	Region 3, C>T 4.09 fold (0.042)
	Dist:	Region 1, T>C 26.17 fold (0.0052)	Dist:	Region 1, T>C 7.19 fold (0.022)		Region 2, T>C 1.56 fold (0.0020)
Aggrecan (*ACAN*)	Prox:	Region 4, C>T 2.53 fold (0.013)	No significant differences		Prox:	Region 1, C>T 1.2 fold (0.014)
Decorin (*DCN*)	Prox:	Region 1, C>T 2.52 fold (0.038)	Prox:	Region 1, C>T 1.57 fold (0.022)	Prox:	Region 1, C>T 2.24 fold (0.010)
Biglycan (*BGN*)	No significant differences		No significant differences		Prox:	Region 4, C>T 2.45 fold (0.031)
Lumican (*LUM*)	Prox:	Region 1, T>C 7.12 fold (0.032)	Prox:	Region 1, T>C 3.52 fold (0.044)	Prox:	Region 4, C>T 3.24 fold (0.034)
	Dist:	Region 1, T>C 13.90 fold (0.0065)	Dist:	Region 1, T>C 5.64 fold (0.0083)		Region 1, T>C 2.91 fold (0.024)
Fibromodulin (*FMOD*)	No significant differences		Prox:	Region 1, C>T 2.00 fold (0.0015)	Prox:	Region 4, C>T 1.80 fold (0.022)
						Region 1, C>T 2.42 fold (0.011)
*TIMP-2*	No significant differences		No significant differences		Prox:	Region 4, C>T 3.96 fold (0.020)
						Region 1, C>T 3.12 fold (0.040)
*TIMP-3*	No significant differences		Prox:	Region 3, C>T 1.53 fold (0.037)	Prox:	Region 4, C>T 1.56 fold (0.040)
						Region 1, C>T 1.89 fold (0.026)
*MMP-9*	No significant differences		No significant differences		Prox:	Region 2, T>C 2.13 fold (0.028)
*MMP-13*	Prox:	Region 1, T>C 102 fold (0.0006)	Prox:	Region 1, T>C 6390 fold (0.020)	Prox:	Region 1, T>C 60 fold (0.0088)
	Dist:	Region 1, T>C 109 fold (0.025)	Dist:	Region 1, T>C 17071 fold (0.0060)	Dist:	Region 1, T>C 83 fold (0.038)
*ADAMTS-4*	No significant differences		Prox:	Region 3, C>T 2.74 fold (0.037)	No significant differences	
				Region 2, C>T 3.09 fold (0.017)		
*ADAMTS-5*	No significant differences		No significant differences		Prox:	Region 4, C>T 3.24 fold (0.014)
*GAPDH*	No significant differences		Prox:	Region 1, C>T 1.55 fold (0.018)	Prox:	Region 4, C>T 1.33 fold (0.039)
						Region 1, C>T 1.42 fold (0.049)

Mean fold changes for each region were calculated by dividing the relative fluorescent unit (RFU) value of transected tendons by the mean RFU of the controls. Differences in gene expression between transected and control tendons were determined using a paired T-test of normalized, log-transformed data to account for pairing of data. P values are presented for each comparison.

#### Control tendons

Consistent with the topographic and temporal differences in histology, multivariate analysis showed effects of time and region on the expression of multiple genes in normal tendons (Tables 1 and 2). Increased expression of *ACAN* (1.15 fold per week post-transection of the contralateral tendon, *P* = 0.022), and *TIMP3* (1.22 fold per week, *P* < 0.001) was seen over time, while *COL3A1* expression decreased (0.89 fold per week, *P* = 0.017). *MMP13* expression tended to decrease with time but this did not achieve significance after correction for false positives (*P* = 0.037). Increased expression of *ADAMTS4* (*P* = 0.031), *TIMP2* (*P* = 0.003), *COL1A1* (*P* = 0.002), and *COL2A1* (*P* = 0.019) were found in control tendons in the distal regions compared to the proximal regions of the tendon, while *MMP13* (*P* < 0.001) and *COL3A1* (*P* < 0.024) expression increased in the tendon in the proximal region compared to the distal region. Expression of *BGN* (*P* < 0.001), *ADAMTS5* (*P* < 0.001), *TIMP-2* (*P* < 0.001), *MMP2* (*P* = 0.028), and *MMP13* (*P* = 0.031) decreased in the control tendons with increasing distance, proximally and distally, from the equivalent site of the lesion in transected tendons.

#### Transected tendons

**Fig 8:**
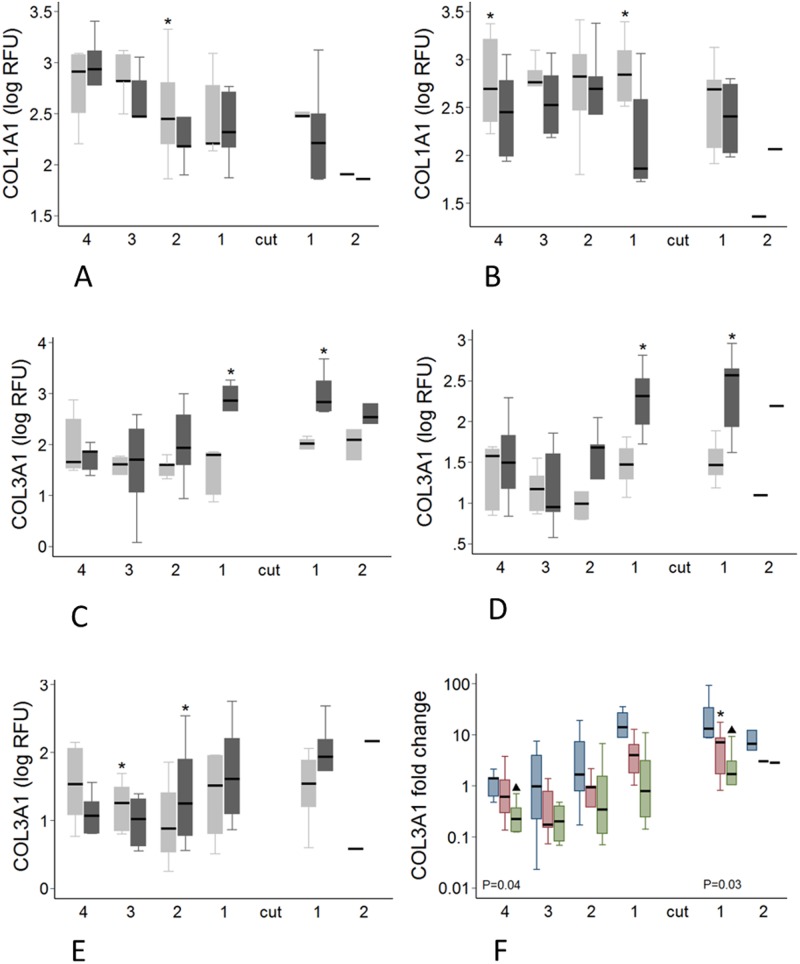
Regional and temporal variation in COL1A1 and COL3A1 expression. A-E: Regional variation in *COL1A1* and *COL3A1* gene expression in transected and control tendons. Transected tendons are represented by the dark grey boxes and control tendons by the light grey boxes. Significant pairwise differences between transected and control tendons (P < 0.05, by paired T-test) are indicated by the asterisk. A-B: *COL1A1* expression at 12 weeks (A) and 16 weeks (B). C-E: *COL3A1* expression at 8(C), 12(D) and 16(E) weeks F: *COL3A1* fold change in transected tendons at 8 weeks (blue boxes), 12 weeks (red boxes) and 16 weeks (green boxes). The P values displayed are for the Kruskal-Wallis analysis where there were significant differences between *COL3A1* expression in the different regions over time. Significant temporal differences between each of the time points in *COL3A1* expression (P < 0.05, as determined by Mann-Whitney U analysis) are indicated by the asterisk (8 weeks significantly different to 12 weeks) and the black triangles (8 weeks significantly different to 16 weeks).

**Fig 9:**
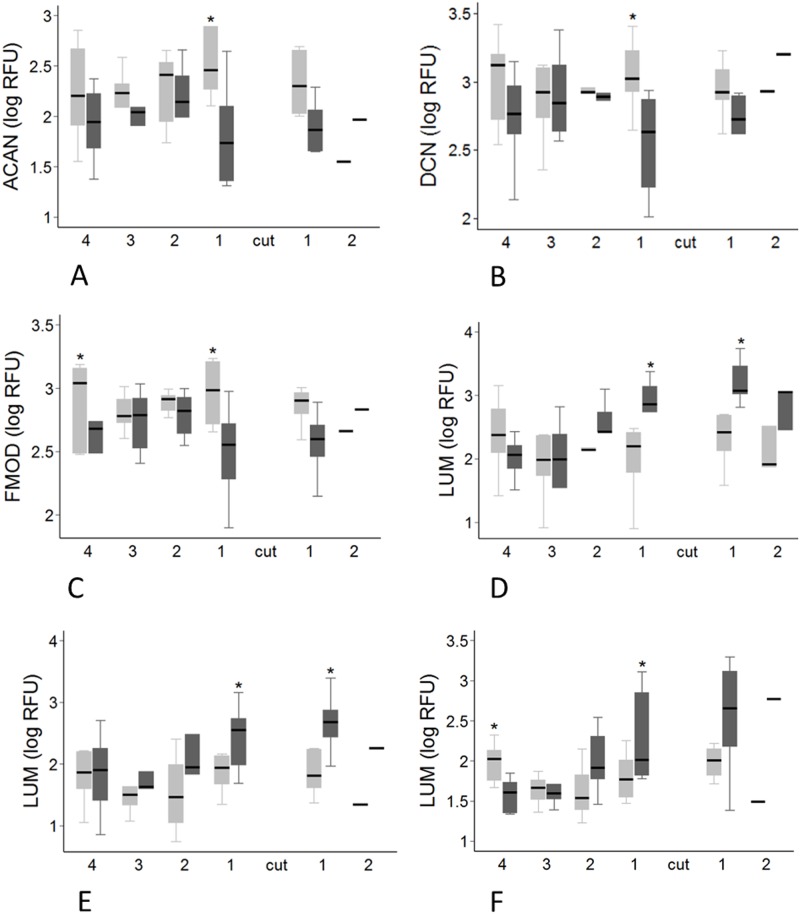
Regional variation in gene expression for proteoglycans. Transected tendons are represented by the dark grey box and control tendons by the light grey box. Significant pairwise differences between transected and control tendons (P < 0.05, by paired t-test) are indicated by the asterisk. A: *ACAN* expression at 16 weeks post-transection. B: *DCN* expression at 16 weeks post-transection. C: *FMOD* expression at 16 weeks post-transection. D-F: *LUM* expression at 8 (D), 12 (E) and 16 weeks (F) post-transection.

**Fig 10:**
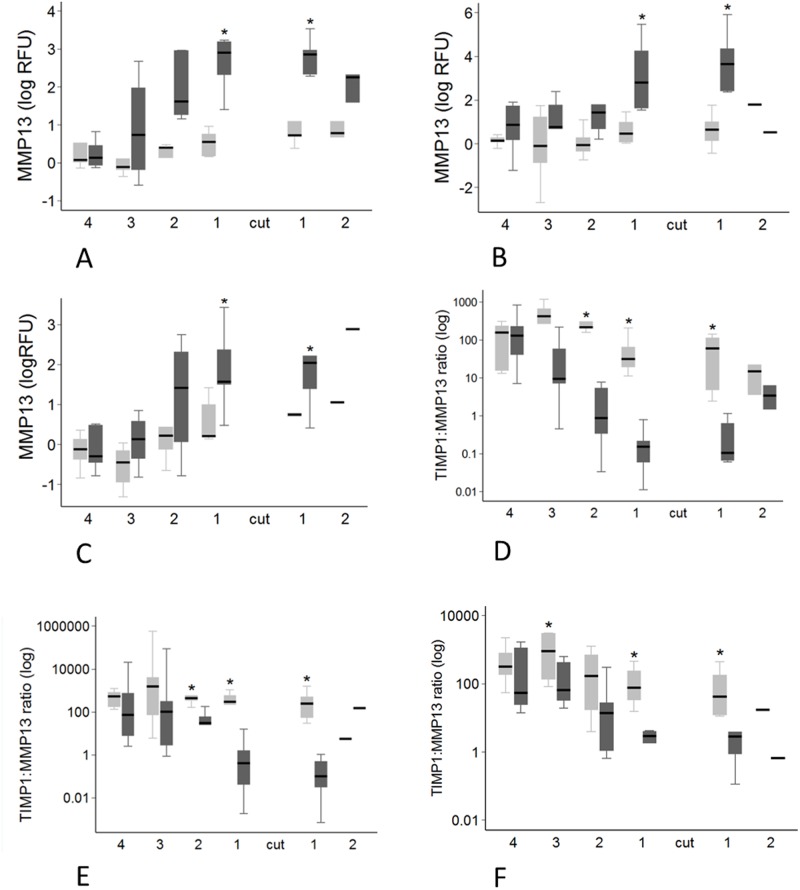
Region variation in *MMP13* expression and *TIMP1*:*MMP13* ratio. Transected tendons are represented by the dark grey box and control tendons by the light grey box. Significant pairwise differences between transected and control tendons (P < 0.05, by paired T-test for *MMP13* expression, and Mann-Whitney U test for *TIMP1*:*MMP13* ratio) are indicated by the asterisk. A-C: *MMP13* expression in at 8 weeks (A), 12 weeks (B) and 16 weeks (C) post-transection. D-F: *TIMP1*:*MMP13* expression ratio at 8 weeks (D), 12 weeks (E) and 16 weeks (F) post-transection.

There was a significant effect of transection on 12 of the 18 genes examined. Decreased expression of *ACAN* (*P* < 0.001), *DCN* (*P* < 0.001), *FMOD* (*P* < 0.001), *TIMP1* (*P* < 0.001), *TIMP2* (*P* < 0.001), *TIMP3* (*P* = 0.001), *COL1A1* (*P* = 0.004), *COL2A1* (*P* < 0.001) and *GAPDH* (P < 0.001) was seen, while expression of *LUM*, *MMP13* and *COL3A1* increased (all *P* < 0.001) (Tables 1 and 2). These effects were not uniform however, with the relative fold change in transected compared with control tendons varying to up to a mean of 17,071 fold depending on the gene, time point and region (Table 3). Significant spatial and temporal differences in the effect of the focal injury on expression of individual genes are summarised in Tables 1 and 2 and shown graphically in Figs 8–10. For most genes, the effect of transection was not altered by time after injury, but in the case of *MMP13* (*P* = 0.027), and *COL3A1* (*P* = 0.005) the changes diminished with time (both decreasing by 0.86 fold per week following transection), while *TIMP3* expression increased 1.22 fold per week (P<0.001).

Changes in expression in transected tendons were greater above the transection for *VCAN* (*P* = 0.007), *LUM* (*P* = 0.017), *MMP13* (*P* = 0.002) and *COL3A1* (*P* = 0.003), while the opposite was seen for *TIMP2* (*P* = 0.028) (Tables 1 and 2). Greater change in expression was seen in proximity to the transection for *BGN* (*P* < 0.001), *LUM* (*P* < 0.001), *ADAMTS5* (*P* < 0.001), *MMP2* (*P* = 0.001), *MMP13* (*P* < 0.001) and *COL3A1* (*P* = 0.002) with expression decreasing progressively with increasing distance from the lesion. In contrast, *COL1A1* expression increased in tendon further removed from the injury site, with gene expression increasing by 1.14 fold per cm from the lesion (P = 0.014) (Tables 1 and 2).

To get a better assessment of the potential remodelling status of the injured tendons, we evaluated the *TIMP1*:*MMP13* ratio as previously suggested [35]. Multivariate analysis showed effects of tendon transection, region and time on the *TIMP1*:*MMP13* ratio. The ratio was decreased in transected tendons, and in the tendon distal to the lesion site compared to the tendon proximal to the lesion site (*P* < 0.001). The ratio was lower close to the lesion site and increased with increasing distance from the lesion site (*P* < 0.001). The ratio generally increased with time after tendon transection (*P* = 0.045). The ratio in transected tendons was lower than control tendons in regions adjacent to the lesion site at 8, 12 and 16 weeks in pairwise analyses (Fig 9D–9F). The *TIMP1*:*MMP13* ratio was only found to be different between time points in region 1 distal to the lesion site in transected tendons (*P* = 0.034). There was an increase in the ratio between 12 and 16 weeks (*P* = 0.036).

#### Immunohistochemistry

**Fig 11:**
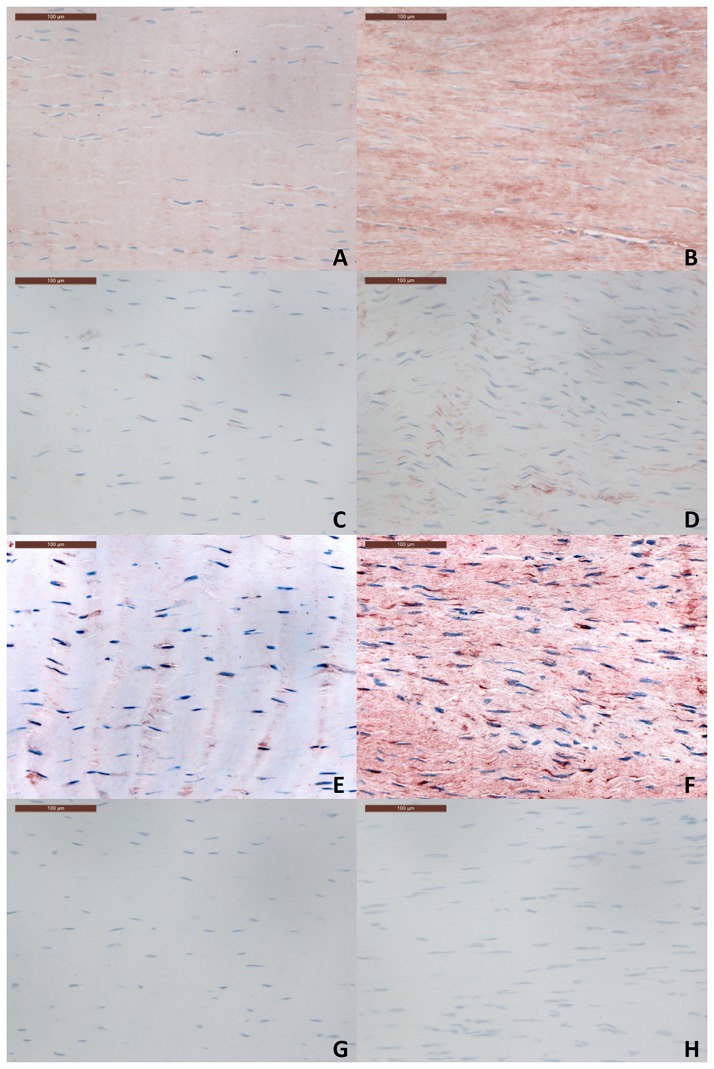
Immunohistochemistry sections for control (A, C, E, G) and transected tendons (B, D, F, H). Representative sections showing collagen III (A-B), MMP13 (C-D) and lumican (E-F) staining are shown. A negative control (G-H) is presented for comparison. The scale bar indicates 0.1mm.

To validate that gene expression results did indicate an increase in protein levels, immunohistochemistry was performed on sections above and below the transection which had gene expression levels approximating the median copy number for the section and time point. Mild immunostaining for collagen III, MMP13 and lumican was noted in control tendons for all proteins. For collagen III this was primarily within the matrix while for MMP13 and lumican, staining was predominantly pericellular. MMP13 staining was noted particularly around blood vessels in control tendons. For all three proteins, an increase in matrix immunostaining in comparison with controls was noted in transected tendons in the regions adjacent to the transection at 8, 12 and 16 weeks (Fig 11). Given that these sections represented the median gene expression level of each gene, increased gene expression levels appeared to reflect increased protein deposition within the extracellular matrix. The increase in immunostaining was marked for collagen III and moderate for MMP13 and lumican (Fig 11) but did not appear to differ between time points. No staining was observed in the negative control sections.

#### Associations between gene expression and histopathology in transected tendons

When all time points were evaluated together, only *COL3A1* and *MMP13* expression correlated with histopathology scores (significance set at *P* ≤ 0.001 by the Benjamini-Hochberg correction). *COL3A1* correlated positively with total pathology score, and specifically increases in cell rounding, cell infiltration and fibre malalignment (*P* ≤ 0.001). While increased *MMP13* was also correlated with an increase in the total score, significant associations with individual parameters were with cellularity and cell infiltration (P ≤ 0.001) (Table 4). When each time point was evaluated in isolation (Tables 5 and 6), a number of significant correlations between expression of particular genes and different histopathology features were identified. The number of correlations between expression of any gene with any histopathological parameter decreased substantially over time in transected tendons: with 27 at week 8, 2 at week 12 and none at 16 weeks after injury. At 8 weeks *COL3A1 ACAN*, *VCAN*, *BGN*, *LUM*, *ADAMTS4*, *MMP2*, *MMP9 and MMP13* correlated positively with one or more histopathological feature, while total pathology at this time significantly positively correlated with *COL3A1 VCAN*, *ADAMTS4*, *MMP9 and MMP13*. At week 12 the only gene significantly correlated with pathology was *ADAMTS4*, both with total score and collagen fibre malalignment.

**Table 4:** Tau-b coefficients and significance of associations between histological scores and gene expression analysis for all transected tendons.

Gene	Sum of Scores	P	Cellularity	P	Cell Morphology	P	Cell Infiltration	P	Alignment	P
*COL3A1*	0.239	**0.0001**	0.227	0.003	0.254	**0.0001**	0.265	**0.001**	0.223	**0.001**
*MMP13*	0.212	**0.001**	0.241	**0.0001**	0.193	0.007	0.234	**0.001**	0.152	0.021

Significance was set at P≤0.001after Benjamini-Hochberg correction. Significant correlations are indicated by the grey boxes.

**Table 5:** Tau-b coefficients and significance of associations between histological scores and gene expression analysis for transected tendons at 8 weeks.

Gene	Sum of Scores	P	Cellularity	P	Cell Morphology	P	Vascularity	P	Cell Infiltration	P	Alignment	P	Proteoglycan	P
*COL3A1*	0.369	**0.0001**	0.419	**0.001**	0.272	**0.009**	0.340	0.014	0.476	**0.0001**	0.330	0.022	0.422	**0.002**
*ACAN*	0.208	0.037	0.171	0.15	0.211	0.046	0.285	**0.009**	0.217	0.081	0.177	0.13	0.303	0.015
*VCAN*	0.341	**0.0001**	0.413	**0.001**	0.248	0.015	0.351	**0.007**	0.469	**0.0001**	0.309	0.025	0.402	**0.004**
*BGN*	0.291	0.014	0.278	0.046	0.272	0.042	0.359	**0.009**	0.323	0.015	0.196	0.15	0.443	**0.002**
*LUM*	0.213	0.052	0.330	**0.01**	0.214	0.045	0.180	0.17	0.203	0.14	0.074	0.59	0.402	**0.004**
*ADAMTS4*	0.273	**0.001**	0.281	**0.005**	0.135	0.13	0.284	0.028	0.372	**0.0001**	0.271	0.019	0.298	0.021
*MMP2*	0.215	0.023	0.169	0.12	0.169	0.2	0.286	0.021	0.218	0.074	0.200	0.079	0.321	**0.008**
*MMP9*	0.230	**0.010**	0.393	<**0.0001**	0.250	0.026	0.146	0.180	0.280	0.013	0.183	0.140	0.375	**0.002**
*MMP13*	0.416	**0.001**	0.418	**0.0001**	0.0260	0.035	0.383	**0.01**	0.457	**0.0001**	0.311	**0.006**	0.321	0.017

Significance was set at P≤0.01 at 8 weeks after Benjamini-Hochberg correction. Significant correlations are indicated by the grey boxes.

**Table 6:** Tau-b coefficients and significance of associations between histological scores and gene expression analysis for transected tendons at 12 weeks.

Gene	Sum of Scores	P	Cellularity	P	Cell Morphology	P	Vascularity	P	Cell Infiltration	P	Alignment	P	Proteoglycan	P
*ADAMTS4*	0.274	**0.001**	0.131	0.21	0.244	0.017	0.217	0.035	0.154	0.18	0.327	**0.001**	0.216	0.041

Significance was set at P≤0.001 at 12 weeks after Benjamini-Hochberg correction. Significant correlations are indicated by the grey boxes.

## Discussion

Tendon pathogenesis is not well understood because there are few experimental, temporal studies following pathology over weeks or months [38–41], and collection of serial, multiple regional samples from damaged tendon in clinical studies without inciting further damage to the tendon is problematic. The current study utilized a partial surgical transection model of tendinopathy and harvested ovine hindlimb superficial flexor tendons 8, 12 and 16 weeks post-transection. Previous studies have used a partial transection model of the ovine infraspinatus tendon [26], and the equine superficial digital flexor tendon to study tendinopathy [22]. Significant findings from these studies included markedly increased histopathology scores and altered gene expression profiles, often distributed widely throughout the tendon. The aim of this study was to expand on these previous studies and provide a comprehensive map of the spatiotemporal changes which occur at the microscopic and molecular level over time, commencing at 8 weeks after tendon injury. Tendon pathology of a lesion after acute injury can be divided into three temporally orchestrated phases which overlap: inflammation, proliferation, and remodelling. However, less is known about the spatiotemporal changes occurring in the tendon regions in close proximity to and remote from the lesion itself. In this study we evaluated these regions and their temporal responses to injury throughout the tendon. We elected to target the remodelling phase which begins at about 6 weeks after injury when the acute inflammatory and proliferative phases are generally complete [18], and when clinical decisions on readiness for return to exercise are made.

A chondroid phenotype is recognised in normal tendons in areas of compression [42], and chondroid metaplasia has also been reported to be a feature in clinical and experimental studies of tendinopathy [21, 43–45]. Aspects of a chondroid phenotype (increased proteoglycan accumulation, cellular rounding and cellularity) were seen in the histology of tendon tissue surrounding the transection throughout the current study, although cellular rounding ameliorated with time. In contrast to previous equine and sheep models of tendinopathy, typical chondroid gene expression changes (including increased expression for *ACAN* and *COL2A1*) [22, 26] were not apparent in the present study. In fact, at all time points, the gene expression for *ACAN* and *COL2A1* was reduced in transected tendons. While this could be associated with differences between specific tendons in the different studies, it likely reflects the longer time after injury (8–16 weeks) in the present study, and that earlier increases in expression induced the chondroid change. A feedback mechanism has been postulated for lumican in tendons, in which a dramatic increase in lumican content may signal to cells to reduce expression of *LUM* mRNA [46]; this mechanism may involve cellular receptor-mediated processes, cellular ‘sensing’ of alterations within the matrix, or upregulation of the JNK pathway [46–48]. A similar mechanism may exist for aggrecan and collagen II.

The other histopathological changes observed in this study are similar to those reported in human tendinopathy [20, 27, 49–52], and previous experimental studies using surgical transection or repetitive exercise models [24, 39]. Our previous studies also found that these histopathological changes were distributed widely throughout the tendon after a focal injury [22]. In the present study, the changes were predominantly localized within the tendon adjacent to the lesion. Some specific features (cell morphology and infiltration) decreased with time, especially after 12 weeks, suggesting reducing inflammation particularly more distant from the injury. This may account for disparity in pathology distribution between studies. However, other aspects of pathology, such as cellularity, proteoglycan accumulation, collagen fibre malalignment and vascularity were not affected by time, suggesting that these tendinopathic alterations are likely to persist long term, with possible implications on structure and function. The presence of neovascularisation seen with ultrasonography has been associated with the clinical symptoms and pain in human Achilles tendinopathy, possibly due to the presence of accompanying nerve development [53]. Although this relationship has been recently questioned [54, 55], minimally invasive tissue stripping techniques that disrupt the new vessels have been suggested to have some efficacy in treatment of Achilles tendinopathy [56]. The persistence of neovascularisation microscopically in our model may suggest mechanisms of ongoing pain or lameness in clinical patients following the initial flexor tendon injury. Further time point studies are warranted to determine the persistence and relevance of these changes in experimentally injured tendons.

Tendons are rarely tested in compression as they are functionally a tensile tissue. Compression was evaluated here in order to determine whether tissue displaying chondroid characteristics was becoming functionally more like cartilage. We found that surgical injury had minimal effect on the compressive modulus of tendon, the only difference detected at 8 weeks following transection in a single region distal to the transection site. It is difficult to reconcile the decrease in compressive modulus in tendon despite the chondroid metaplasia, but this may be associated with the analysis method. A previous study of bovine femoral articular cartilage tested in unconfined compression demonstrated an average modulus of 0.31+/-0.18 MPa at 20% strain which is comparable to our results in tendon [57]. We tested the tendons in transverse compression with the force directed perpendicular to the fibre orientation. Tendon tissue has different mechanical properties depending on fibre orientation [58], and testing with the force directed parallel to the fibre direction may have yielded different results.

In the current study changes in gene expression and histopathology were often localized, adjacent to the lesion. This is in contrast to a previous study that found widespread gene expression changes in a similar surgical transection model of tendinopathy in horses, 6 weeks following injury [22]. The differences between these two studies may reflect the later time points in the present study. The process of tendon repair is initiated by the inflammatory response to injury at the site of the lesion [18], and components of the inflammatory response have been shown to be active in both the acute and chronic phases of naturally-occurring equine tendinopathy [59]. This is consistent with the findings in the current study, in which a marked increase in inflammatory cell infiltration score was seen in transected tendons which reduced over time (S3 Table). Additionally, the expression of MMP-13 (the upregulation of which has previously been associated with inflammation in tendons) [60, 61] progressively decreased and localized over time (Table 2, Fig 9).The mechanism underlying the changes seen in gene expression and histology appears to reflect the resolution and localization of the inflammatory process. However, temporal changes in biomechanical influences may also play a role [22, 26] as repair of the lesion and changes in diameter restore tendon mechanical properties. It is likely that an initial, widespread increase in cellular synthetic activity associated with the inflammatory response to injury and changes in biomechanics diminishes over time, resulting in the ‘retraction’ of the widespread changes in gene expression and histology to the areas surrounding the lesion. In addition to the inflammatory response, other sources of synthetically active cells (such as those derived from the tendon wound edges, surrounding tissues or circulation) may be implicated in the healing process [18, 62–64]. Given the focal proliferation and adhesion of the paratenon noted in the region of the healing lesion (which has also been observed in naturally-occurring tendinopathy [59]), the possibility of reparative progenitor cells derived from the paratenon cannot be excluded. This population of cells existing in close proximity to the lesion site may contribute to the localized changes seen, and represent a significant mechanism for tendon repair.

Regardless of the underlying mechanism, the changes in gene expression and histopathology observed in the current model provide some insight into the response of tendon to injury and the pathophysiology of tendinopathy over time. Twenty-seven positive correlations between gene expression and histopathology were seen at 8 weeks post-injury, but only two at 12 weeks and none at 16 weeks after injury. This suggests that early gene expression changes may drive the development of subsequent pathological features. Eight weeks after partial surgical transection there was in an increase in the expression of *COL3A1*, *MMP13*, and *LUM*. These increases were sustained regionally in injured tendons for the duration of the study despite a progressive general reduction in *COL3A1* and *MMP13* expression. The presence of increased collagen III, MMP13 and lumican in transected tendons in comparison to controls (in the sections above and below the transection) was also noted on immunohistochemistry at all time points, indicating that gene expression changes were indicative of absolute protein levels. Collagen III is upregulated in the initial phases of tendon injury [40], and increases in expression and changes in collagen III:I ratio have been observed both in patients and other experimental studies [22, 30, 65, 66]. The positive association with cell rounding and cellular infiltration suggests that the increase in *COL3A1* expression may be produced by the pathological rounded cells and infiltrating interfibrillar cells observed histologically, early in the healing process. This is supported by the lack of a significant correlation between *COL3A1* expression and cell morphology beyond 8 weeks. In normal tendon, collagen III is typically interposed with collagen I, and is described as producing smaller, weaker fibres [67]. In injured tendon, increased expression of *COL3A1* occurred adjacent to the lesion site, which, in combination with a concurrent regional reduction in *COL1A1* expression, may produce weaker collagen fibres locally. The positive correlation of *COL3A1* expression with collagen fibre malalignment also suggests a possible role in production of functionally abnormal tissue. This healing response is consistent with biomechanical studies that suggest recurrent injury of the equine superficial digital flexor tendon is most likely to occur in the region adjacent to the original injury [23].

Upregulation of the collagenase MMP13 in tendon injury has been reported in other experimental models. In addition to the previously-discussed association with inflammation, its expression appears to be influenced by the biomechanical environment [68–70]. Upregulation is thought to be a catabolic response by resident tenocytes to a loss of homeostatic tension [37, 70]. This is postulated to occur as a result of microscopic collagen fibre damage leading to localized areas of stress deprivation, as well as overloading [37, 71]. Persistent upregulation of MMP13 after injury could negatively affect tendon strength by cleaving collagen I during the healing period. MMP13 is the principal collagenase upregulated in tendinopathy of the human rotator cuff and has been implicated in the progression of partial tears into full thickness tears [68, 69, 72]. In the current study regional stress deprivation in the partially transected tendons throughout the 16 week period may have been responsible for the upregulation of *MMP13* expression, and contributed to ongoing collagenolysis and a tendinopathic state in the healing tendon [68]. The positive correlation between *MMP13* expression and collagen fibre malalignment at 8 weeks suggests a state of collagenolytic remodelling may develop early. However, given the positive correlation of *MMP13* expression with cellularity and cell infiltration in transected tendons, infiltrating cells (in addition to native cells in an abnormal biomechanical environment) may also be responsible for the increase in *MMP13* mRNA production in the period up to 8 weeks after injury. The gradual reduction in *MMP13* expression levels over the study period is consistent with that seen in a previous study of ligament injury [73]. The overall decrease in *MMP13* expression in the later stages of injury may reflect a return of homeostatic tension as the repair tissue matures, or re-establishment of a homeostatic set-point by contractile tendon cells [70].

*TIMP1*, *2* and *3* expression was decreased in transected tendons. TIMPs inhibit the catabolic activities of MMPs by binding to them in a reversible 1:1 stoichiometric relationship [74]. A general decrease in *TIMP* expression levels combined with upregulation of *MMP13* expression suggests a potentiated state of MMP-mediated matrix catabolism in transected tendons up to 16 weeks after injury. While lower than controls, *TIMP3* expression increased over time in transected tendons. Apart from inhibition of metalloproteinases, particularly the ADAMTS proteoglycanases, TIMP3 has anti-angiogenic properties, and its presence in the extracellular matrix may prevent blood vessel invasion by preventing the proteolytic release of angiogenic factors [75]. The increase in *TIMP3* expression in transected tendons with time post-injury may act to moderate neovascularization as healing progresses.

An imbalance in the ratio between MMPs and TIMPs is reported to play a role in the development of pathology within tendons and is a suggested cause of histological collagenolysis [36, 37]. There was a significant imbalance in the *TIMP1*:*MMP13* ratio in this study, most notably a reduced ratio adjacent to the lesion in transected tendons. The decrease in the ratio was driven by both a marked increase in *MMP13* and a reduction in *TIMP1* expression in transected tendons. While still requiring post-translational activation, the decrease in *TIMP1*:*MMP13* may allow for increased matrix catabolism and active collagenolysis around the area of the injury, even at 16 weeks post-injury. This may contribute to reinjury in this region rather than the healed injury site itself [23]. Both MMP inhibitors and upregulation of TIMP1 (with controlled exercise) have been suggested as having potential in the treatment of tendon disorders [37, 76, 77]. Considering the marked negative effects of injury on *TIMP1*:*MMP13* ratio in combination with the continued regional upregulation of *MMP13* at 16 weeks in transected tendons, a prolonged therapeutic course with MMP inhibitors (possibly in combination with a controlled exercise program) may represent a possible avenue for future treatment of tendinopathy. However, the early positive correlation of *MMP13* expression with several histopathological features at 8 weeks post-injury in combination with a large number of positive correlations between other genes and histopathology at this time point suggests that the molecular mechanisms promoting pathology are present early in the course of healing. Any future therapies targeting the molecular mechanisms of tendinopathy may have most benefit if commenced in the preliminary stages of healing prior to the deposition and establishment of abnormal matrix.

Regionally *LUM* expression was upregulated at all time points in transected tendons, particularly in the regions directly adjacent to the lesion. At 8 weeks, LUM along with *VCAN* and *BGN*, was also positively correlated with proteoglycan score. This suggests that lumican deposition along with other proteoglycans, may contribute to the increased proteoglycan staining seen in transected tendons. Increased *LUM* expression has been reported in stress deprived areas in other tendinopathy studies [22, 26]. Lumican has a well-recognized role in normal collagen fibril assembly [78], and a possible role in collagen fibril malalignment in pathological tendon [22]. Lumican may also reduce the susceptibility of type I collagen fibres to the collagenases, MMP1 and MMP13, and in itself, is minimally degraded by MMP13 [79, 80]. The increase in *LUM* expression seen in the current study may therefore represent a protective response in injured tendons. However, lumican also competes with fibromodulin for its collagen-binding site on the collagen fibril [81, 82]. It is suggested that both lumican and fibromodulin can create cross-links between collagen fibrils, but in comparison with fibromodulin those mediated by lumican may result in a mechanically weaker fibril [82]. Furthermore, a lack of fibromodulin has been associated with the formation of thinner collagen fibrils and abnormal tendon fibres with an accumulation of lumican [46, 82]. In the present study, a reduction in *FMOD* expression was seen in region 1 directly proximal to the lesion in transected tendons at both 12 and 16 weeks. This, combined with the consistent, regional upregulation of *LUM* at all time points in the two regions directly adjacent to the lesion may contribute to the collagen fibrils present in the repair tissue adjacent to the lesion being weaker, which could contribute to re-injury in this area.

*DCN* expression was regionally decreased immediately proximal to the lesion site at all time points. Whether decorin has the same role in healing tendon as it does in the developing tendon is unclear [83]. *In vivo*, the use of decorin antisense therapy to limit *DCN* expression within healing ligaments resulted in thicker collagen fibres with improved mechanical properties [84]. Additionally, it may suppress the production of transforming growth factor-β1, a major mediator of tissue fibrosis [85]. Augmenting the natural down-regulation of decorin production may provide an avenue for treatment of acute tendon injuries.

We identified a number of other concurrent regional variations in gene expression over time. *BGN*, *ADAMTS5*, *MMP2*, and *MMP13* expression was decreased with increasing distance from the lesion site, in both transected and control tendons, while *MMP13*, *TIMP2* and *COL3A1* showed similar proximal or distal distribution of expression in both transected and control tendons. This commonality supports the suggestion that gene expression of some matrix components by tenocytes is regulated by common biomechanical factors within the regional tendon environment [86]. In transected tendons, *COL1A1* expression increased while *COL3A1* was decreased with increasing distance from the lesion. It is likely that local humoral factors or cytokines released from the lesion itself also play a part in regulation of gene expression by tenocytes in injured tendon, but the exact role of these factors in tendon disorders is still unclear [87]. However, where isolated regional gene expression differences between transected and control tendons existed (such as for *COL1A1*, *ACAN*, *BGN*, *TIMP2*, *TIMP3*, *ADAMTS4*, *ADAMTS5* and *GAPDH*) (Table 3), no consistent trends or patterns were observed at the different time points. It is possible that some changes seen may be incidental and have little biological relevance despite being statistically significant.

In contrast, consistent trends in expression were seen for *COL3A1*, *LUM* and *MMP13* at all time points and these changes are considered highly likely to be biologically significant, especially considering the immunohistochemical findings. The sustained increase in expression and deposition of *COL3A1*, *LUM*, and *MMP13* in the regions adjacent to the lesion, combined with a regional decrease in *DCN* expression seen throughout the study period, demonstrates ongoing disturbances in tissue metabolism and structure for up to 16 weeks following surgical partial transection of the superficial flexor tendons in this model of tendinopathy. In horses and humans with clinical tendon injury or tendinopathy, rehabilitation programs following tendon injury often recommend a graded increase in or a return to normal activity within 16 weeks after tendon injury depending on clinical progress and/or the results of ultrasonographic examination [88, 89]. However, ultrasound has been shown to underestimate the degree of pathology occurring at the molecular and structural levels in an equine model of tendinopathy [22]. The altered gene expression profile and tissue architecture adjacent to the original lesion seen at 16 weeks post-injury in the current study would suggest these programs should be approached with caution, especially in view of studies which indicate a higher recurrence of tendinopathy after an early return to athletic activity [90–92].

In addition to the findings in transected tendons, significant temporal effects were also identified in control tendons: reduced cell rounding, significantly decreased *COL3A1* expression, a trend for decreasing *MMP13* expression, and increasing *TIMP3* and *ACAN* expression. Similarly, positive correlations between *ACAN* and *COL2A1* expression with proteoglycan score were seen in control tendons, suggesting an increase in chondroid characteristics in the uninjured tendon. The presence of some similar temporal findings in transected and control tendons (e.g. changes in *MMP13* and *COL3A1* expression) may suggest continued maturational change despite the animals being skeletally mature. Alternatively the use of contralateral limbs as controls may confound the findings. Previous studies have found bilateral changes in unilateral tendinopathy models and in humans with Achilles tendinopathy [32, 33], and suggested involvement of central neuronal mechanisms in the pathophysiology of tendon disease [93]. Clinically, bilateral tendinopathy frequently occurs in humans [10, 16]. Patients who have suffered an Achilles tendon rupture are almost 200 times more likely to rupture the Achilles tendon of the opposite limb [17], and bilateral clinical improvement has been observed in patients with Achilles tendinopathy after unilateral surgical treatment, although the mechanisms were not elucidated [94]. It is interesting to speculate whether unilateral injury in our model may result in structural or molecular changes in the contralateral limb, either as a result of altered biomechanical forces, systemic influences or neuronal mechanisms. However, we found no difference in gene expression between sham-operated and contralateral limbs, suggesting the temporal changes identified are driven systemically.

There were several limitations to this study. The suitability of a surgical transection model of tendinopathy has been questioned as a model for the naturally occurring condition. Clinically, tendinopathy is often considered to be an overuse injury so it has been suggested that a surgical transection model is an inappropriate model for drawing conclusions [39]. However this study and others have identified molecular and histopathologic changes in transected tendons typical of overuse tendinopathy, supporting the utility of the model, particularly for the study of temporal changes occurring after injury [24, 95]. Gene expression changes may not correspond to absolute protein levels or *de novo* synthesis due to post-translational modification or other mechanisms. However, as other authors have noted, it is reasonable to postulate that alterations in gene expression are likely to represent differing extracellular environments, with possible effects on tendon structure and function [25]. In addition, our major gene expression findings (regional increases in *COL3A1*, *MMP13* and *LUM*) were consistent with increased immunohistochemical staining, suggesting that in this particular study, gene expression levels are likely to be indicative of increased tissue protein synthesis. This study may also have been limited by the regional nature of sampling. Only the lateral side of the tendon was examined for gene expression and histological changes, and only the medial side for biomechanical analyses. Some studies have indicated differential gene expression patterns for overstressed and understressed regions [22, 26]. It is possible that we may have underestimated the degree of change by not testing both lateral and medial sides of the tendon for gene expression, histological or biomechanical differences. Unfortunately, the small size of the sheep tendons precluded analysis of both over and under-stressed regions [22]. The position of the lesion within the tendon precluded equal samples being retrieved above and below the lesion, and the most distal samples were collected very close to the insertion of the tendon. Insertional regions are known to experience higher compression forces and often have fibrocartilaginous characteristics [40, 96]. The significant effects of a distal location on gene expression, histopathology and biomechanics may be due in some part to the physical position within the tendon rather than an effect of surgical injury. Finally, no naïve control tendons were analyzed at any time point; an effect of age and continued maturational change cannot be ruled out as a cause of the histological and molecular changes seen in contralateral tendons. This experimental model may have some utility for bilateral studies and studies of the contralateral limb in tendinopathy, however a larger population of independent, non-operated controls are needed to determine whether the temporal changes we observed are occurring routinely in normal tendons in naïve animals, or whether there is a direct effect of even mild injury (sham-surgery) on the contralateral limb.

## Conclusions

This study highlights the complex environment of healing tendon during the remodelling phase following partial tendon transection. There are notable variations in gene expression and histological parameters between regions as well as intra-regional variation between time points. One aspect of the chondroid phenotype (cellular rounding) was noted to decrease over time, but time did not have any effect on cellularity, vascularity or proteoglycan accumulation. The presence of regionally sustained *COL3A1*, *MMP13*, and *LUM* expression, persistently decreased *TIMP1*:*MMP13* ratio, and decreased *DCN* expression indicates that derangements in tendon matrix metabolism are still occurring as late as 16 weeks post-injury, especially close to the primary lesion. Furthermore the study suggested the possibility of a bilateral effect of an isolated unilateral tendon injury. These findings should be considered when formulating clinical rehabilitation plans following tendon injury. The identified changes raise the potential for novel therapeutic approaches, such as metalloproteinase inhibition or genetic manipulation technologies; additionally, if these therapies are considered, a prolonged course of treatment (up to or beyond 16 weeks) may be justified. To provide more comprehensive understanding of the process of tendon healing in this model, further time point studies including naïve controls are warranted at 6–12 months post-injury to evaluate the manner of tendon healing in the longer term.

## Supporting information

S1 Table Analysed genes and relevant primers. Analysed genes (with standard abbreviations as used in the text), gene sequence accession numbers, primer sequences, annealing temperatures and product sizes are shown.
(DOCX)

S2 Table Histology scores and descriptions. Histology scoring parameters are shown. Slides were scored by three separate observers and a mean score calculated. The total histopathology score was calculated by adding all scores (except the proteoglycan score). A higher score indicated a tendon with a more pathological appearance.
(DOCX)

S3 Table Histology scoring odds ratios for spatiotemporal covariates with and without surgery by mixed ordinal logistic regression. Ratios are presented for non-operated controls (contralateral tendons and sham operated) and for both operated and non-operated tendons. CI = confidence intervals; 5% significance level is P<0.025 after Benjamini-Hochberg correction.
(DOCX)

S1 Spreadsheet Dataset with raw data for all gene expression, biomechanics and histology analyses. (XLSX)

## References

[1] Moller A, Astrom M, Westlin NE. Increasing incidence of Achilles tendon rupture. Acta Orthop Scand. 1996; 67: 479–81. 8948254 10.3109/17453679608996672

[2] Maffulli N, Waterston SW, Squair J, Reaper J, Douglas AS. Changing incidence of Achilles tendon rupture in Scotland, a 15-year study. Clin J Sport Med. 1999; 9: 157–60. 10512344 10.1097/00042752-199907000-00007

[3] Leppilahti J, Puranen J, Orava S. Incidence of Achilles tendon rupture. Acta Orthop Scand. 1996; 67: 277–9. 8686468 10.3109/17453679608994688

[4] Leppilahti J, Orava S. Total Achilles tendon rupture—A review. Sports Med. 1998; 25: 79–100. 9519398 10.2165/00007256-199825020-00002

[5] Longo UG, Ronga M, Maffulli N. Achilles Tendinopathy. Sports Med Arthrosc. 2009; 17: 112–26. doi: 10.1097/JSA.0b013e3181a3d625 19440139 10.1097/JSA.0b013e3181a3d625

[6] Kujala UM, Sarna S, Kaprio J. Cumulative incidence of Achilles tendon rupture and tendinopathy in male former elite athletes. Clin J Sport Med. 2005; 15: 133–5. 15867554 10.1097/01.jsm.0000165347.55638.23

[7] Kvist M. Achilles tendon injuries in athletes. Sports Med. 1994; 18: 173–201. 7809555 10.2165/00007256-199418030-00004

[8] de Jonge S, van den Berg C, de Vos RJ, van der Heide HJL, Weir A, Verhaar JAN, et al Incidence of midportion Achilles tendinopathy in the general population. Brit J Sport Med. 2011; 45: 1026–8. 10.1136/bjsports-2011-090342 21926076

[9] Albers IS, Zwerver J, Diercks RL, Dekker JH, Van den Akker-Scheek I. Incidence and prevalence of lower extremity tendinopathy in a Dutch general practice population: a cross sectional study. BMC Musculoskelet Disord. 2016; 17: 16–21. doi: 10.1186/s12891-016-0885-2 26759254 10.1186/s12891-016-0885-2 PMC4711046

[10] Jarvinen TA, Kannus P, Maffulli N, Khan KM. Achilles tendon disorders: etiology and epidemiology. Foot Ankle. 2005; 10: 255–66. 10.1016/j.fcl.2005.01.013 15922917

[11] Kasashima Y, Takahashi T, Smith RKW, Goodship AE, Kuwano A, Ueno T, et al Prevalence of superficial digital flexor tendonitis and suspensory desmitis in Japanese Thoroughbred flat racehorses in 1999. Equine Vet J. 2004; 36: 346–350. 15163043 10.2746/0425164044890580

[12] Avella CS, Ely ER, Verheyen KLP, Price JS, Wood JLN, Smith RKW. Ultrasonographic assessment of the superficial digital flexor tendons of National Hunt racehorses in training over two racing seasons. Equine Vet J. 2009; 41: 449–54. 19642404 10.2746/042516409x391042

[13] Marr CM, Marr CM, Love S, Boyd JS, McKellar Q. Factors affecting the clinical outcome of injuries to the superficial digital flexor tendon in National Hunt and point-to-point racehorses. Vet Rec. 1993; 132: 476–479 8506599 10.1136/vr.132.19.476

[14] O'Meara B, Bladon B, Parkin TDH, Fraser B, Lischer CJ. An investigation of the relationship between race performance and superficial digital flexor tendonitis in the Thoroughbred racehorse. Equine Vet J. 2010; 42: 322–6. doi: 10.1111/j.2042-3306.2009.00021.x 20525050 10.1111/j.2042-3306.2009.00021.x

[15] Wilkins R, Bisson LJ. Operative Versus Nonoperative Management of Acute Achilles Tendon Ruptures A Quantitative Systematic Review of Randomized Controlled Trials. Am J Sports Med. 2012; 40: 2154–60. doi: 10.1177/0363546512453293 22802271 10.1177/0363546512453293

[16] Paavola M, Kannus P, Paakkala T, Pasanen M, Jarvinen M. Long-term prognosis of patients with Achilles tendinopathy—An observational 8-year follow-up study. Am J Sports Med. 2000; 28: 634–42. 11032217 10.1177/03635465000280050301

[17] Aroen A, Helgo D, Granlund OG, Bahr R. Contralateral tendon rupture risk is increased in individuals with a previous Achilles tendon rupture. Scand Journal Med Sci Spor. 2004; 14: 30–3. 10.1111/j.1600-0838.2004.00344.x 14723785

[18] Sharma P, Maffulli N. Biology of tendon injury: healing, modeling and remodeling. J Musculoskelet Neuronal Interact. 2006; 6: 181–90. 16849830

[19] Rees J MN, Cook J. Management of Tendinopathy. Am J Sports Med. 2009; 37: 1855–1867. doi: 10.1177/0363546508324283 19188560 10.1177/0363546508324283

[20] Astrom M, Rausing A. Chronic Achilles tendinopathy–a survey of surgical and histopathologic findings. Clin Orthop Relat R. 1995: 151–64. 7634699

[21] Hashimoto T, Nobuhara K, Hamada T. Pathologic evidence of degeneration as a primary cause of rotator cuff tear. Clin Orthop Relat R. 2003: 111–20. 10.1097/01.blo.0000092974.12414.22 14612637

[22] Jacobsen E, Dart AJ, Mondori T, Horadogoda N, Jeffcott LB, Little CB, et al Focal experimental injury leads to widespread gene expression and histologic changes in equine flexor tendons. Plos One. 2015; 10(4): e0122220 doi: 10.1371/journal.pone.0122220 25837713 10.1371/journal.pone.0122220 PMC4383631

[23] Crevier-Denoix N, Collobert C, Pourcelot P, Denoix JM, Sanaa M, Geiger D, et al Mechanical properties of pathological equine superficial digital flexor tendons. Equine Vet J. 1997; 29: 23–6. 10.1111/j.2042-3306.1997.tb05046.x 9354282

[24] Cadby JA, David F, van de Lest C, Bosch G, van Weeren PR, Snedeker JG, et al Further characterisation of an experimental model of tendinopathy in the horse. Equine Vet J. 2013; 45: 642–8. doi: 10.1111/evj.12035 23448172 10.1111/evj.12035

[25] Jones GC C A, Pennington CJ, Clark IM, Edwards DR, Bradley MM, Hazleman BL, Riley GP. Expression profiling of metalloproteinases and tissue inhibitors of metalloproteinases in normal and degenerate human Achilles tendon. Arthritis Rheum. 2006; 54: 832–842. doi: 10.1002/art.21672 16508964 10.1002/art.21672

[26] Smith MM S G, Sakurai G, Smith SM, Young AA, Melrose J, Stewart CM, et al Modulation of aggrecan and ADAMTS expression in ovine tendinopathy Induced by altered strain. Arthritis Rheum. 2008; 58: 1055–66. doi: 10.1002/art.23388 18383380 10.1002/art.23388

[27] de Mos M, van El B, DeGroot J, Jahr H, van Schie HTM, van Arkel ER, et al Achilles tendinosis—Changes in biochemical composition and collagen turnover rate. Am J Sport Med. 2007; 35: 1549–56. 10.1177/0363546507301885 17478653

[28] Karousou E, Ronga M, Vigetti D, Passi A, Maffulli N. Collagens, proteoglycans, MMP-2, MMP-9 and TIMPs in human achilles tendon rupture. Clin Orthop Relat R. 2008; 466: 1577–82. 10.1007/s11999-008-0255-y PMC2505242 18425559

[29] Nomura M, Hosaka Y, Kasashima Y, Ueda H, Takehana K, Kuwano A, et al Active expression of matrix metalloproteinase-13 mRNA in the granulation tissue of equine superficial digital flexor tendinitis. J Vet Med Sci. 2007; 69: 637–9. 17611361 10.1292/jvms.69.637

[30] Ireland D, Harrall R, Curry V, Holloway G, Hackney R, Hazleman B, et al Multiple changes in gene expression in chronic human Achilles tendinopathy. Matrix Biol. 2001; 20: 159–69. 11420148 10.1016/s0945-053x(01)00128-7

[31] Choi RK, Smith MM, Martin JH, Clarke JL, Dart AJ, Little CB, et al Chondroitin sulphate glycosaminoglycans contribute to widespread inferior biomechanics in tendon after focal injury. J Biomech. 2016; 49: 2694–701. doi: 10.1016/j.jbiomech.2016.06.006 27316761 10.1016/j.jbiomech.2016.06.006

[32] O'Brien EJO, Shrive NG, Rosvold JM, Thornton GM, Frank CB, Hart DA. Tendon mineralization is accelerated bilaterally and creep of contralateral tendons is increased after unilateral needle injury of murine achilles tendons. J Orthopaed Res. 2013; 31:1520–8. 10.1002/jor.22404 23754538

[33] Docking SI, Rosengarten SD, Daffy J, Cook J. Structural integrity is decreased in both Achilles tendons in people with unilateral Achilles tendinopathy. J Sci Med Sport. 2015; 18: 383–7. doi: 10.1016/j.jsams.2014.06.004 25024134 10.1016/j.jsams.2014.06.004

[34] McCurdy RD, McGrath JJ, Mackay-Sim A. Validation of the comparative quantification method of real-time PCR analysis and a cautionary tale of housekeeping gene selection. Gene Ther Mol Biol. 2008; 12:15–24.

[35] Ayers D, Clements DN, Salway F, Day PJ. Expression stability of commonly used reference genes in canine articular connective tissues. BMC Vet Res. 2007; 3: 7–17. doi: 10.1186/1746-6148-3-7 17484782 10.1186/1746-6148-3-7 PMC1884148

[36] Fu SC, Chan BP, Wang W, Pau HM, Chan KM, Rolf CG. Increased expression of matrix metalloproteinase 1 (MMP1) in 11 patients with patellar tendinosis. Acta Orthop Scand. 2002; 73: 658–62. doi: 10.1080/000164702321039624 12553513 10.1080/000164702321039624

[37] Gardner K, Arnoczky SP, Caballero O, Lavagnino M. The effect of stress-deprivation and cyclic loading on the TIMP/MMP ratio in tendon cells: an in vitro experimental study. Disabil Rehabil. 2008; 30: 1523–9. doi: 10.1080/09638280701785395 18665569 10.1080/09638280701785395

[38] Soslowsky LJ, Thomopoulos S, Tun S, Flanagan CL, Keefer CC, Mastaw J, et al Neer Award 1999—Overuse activity injures the supraspinatus tendon in an animal model: A histologic and biomechanical study. J Shoulder Elb Surg. 2000; 9: 79–84. 10810684

[39] Cho NS, Hwang JH, Lee YT, Chae SW. Tendinosis-like histologic and molecular changes of the Achilles tendon to repetitive stress: A pilot study in rats. Clin Orthop Related R. 2011; 469: 3172–80. 10.1007/s11999-011-2000-1 PMC3183207 21800208

[40] Dahlgren LA, Brower-Toland BD, Nixon AJ. Cloning and expression of type III collagen in normal and injured tendons of horses. Am J Vet Res. 2005; 66: 266–70. 15757126 10.2460/ajvr.2005.66.266

[41] Schramme M, Hunter S, Campbell N, Blikslager A, Smith R. A surgical tendonitis model in horses: Techinque, clinical, ultrasonographic and histological characterisation. Vet Comp Orthopaed. 2010; 23: 231–9. 10.3415/VCOT-09-10-0106 20585715

[42] Benjamin M. The structure and function of tendons In: Hazleman B, Riley G, Speed C, editors. Soft Tissue Rheumatology. Oxford: Oxford University Press; 2004.

[43] Lui PPY, Fu SC, Chan LS, Hung LK, Chan KM. Chondrocyte phenotype and ectopic ossification in collagenase-induced tendon degeneration. J Histochem Cytochem. 2009; 57: 91–100. doi: 10.1369/jhc.2008.952143 18824634 10.1369/jhc.2008.952143 PMC2628327

[44] de Mos M, Koevoet W, van Schie HTM, Kops N, Jahr H, Verhaar JAN, et al In vitro model to study chondrogenic differentiation in tendinopathy. Am J Sports Med. 2009; 37: 1214–22. doi: 10.1177/0363546508331137 19279223 10.1177/0363546508331137

[45] Archambault JM, Jelinsky SA, Lake SP, Hill AA, Glaser DL, Soslowsky LJ. Rat supraspinatus tendon expresses cartilage markers with overuse. J Orthopaed Res. 2007; 25: 617–24. 10.1002/jor.20347 17318892

[46] Svensson L, Aszódi A, Reinholt FP, Fässler R, Heinegård D, Oldberg Å. Fibromodulin-null mice have abnormal collagen fibrils, tissue organization, and altered lumican deposition in tendon. J Biol Chem. 1999; 274: 9636–47. 10092650 10.1074/jbc.274.14.9636

[47] Chakravarti S. Functions of lumican and fibromodulin: Lessons from knockout mice. Glycoconjugate J. 2002; 19: 287–93. 10.1023/A:1025348417078 12975607

[48] Chen J, Wong-Chong J, SundarRaj N. FGF-2– and TGF-β1–Induced Downregulation of Lumican and Keratocan in Activated Corneal Keratocytes by JNK Signaling Pathway. Invest Ophth Vis Sci. 2011; 52: 8957–64. 10.1167/iovs.11-8078 PMC3231797 22025571

[49] Kannus P, Jozsa L. Histopathological changes preceding spontaneous rupture of a tendon–a controlled study of 891 patients. J Bone Joint Surg Am. 1991; 73A: 1507–25. 1748700

[50] Tallon C, Maffulli N, Ewen SWB. Ruptured Achilles tendons are significantly more degenerated than tendinopathic tendons. Med Sci Sport Exer. 2001; 33: 1983–90. 10.1097/00005768-200112000-00002 11740288

[51] Jelinsky SA, Rodeo SA, Li J, Gulotta LV, Archambault JM, Seeherman HJ. Regulation of gene expression in human tendinopathy. BMC Musculoskelet Disord. 2011; 12: 86–97. doi: 10.1186/1471-2474-12-86 21539748 10.1186/1471-2474-12-86 PMC3095578

[52] Corps AN, Robinson AHN, Harrall RL, Avery NC, Curry VA, Hazleman BL, et al Changes in matrix protein biochemistry and the expression of mRNA encoding matrix proteins and metalloproteinases in posterior tibialis tendinopathy. Ann Rheum Dis. 2012; 71: 746–52. doi: 10.1136/annrheumdis-2011-200391 22241901 10.1136/annrheumdis-2011-200391 PMC3329235

[53] Ohberg L, Lorentzon R, Alfredson H. Neovascularisation in Achilles tendons with painful tendinosis but not in normal tendons: an ultrasonographic investigation. Knee Surg Sport Tra A. 2001; 9: 233–8. 10.1007/s001670000189 11522081

[54] De Jonge S, Warnaars JLF, De Vos RJ, Weir A, van Schie HTM, Bierma-Zeinstra SMA, et al Relationship between neovascularization and clinical severity in Achilles tendinopathy in 556 paired measurements. Scand J Med Sci Spor. 2014; 24: 773–8. 10.1111/sms.12072 23600756

[55] Tol JL, Spiezia F, Maffulli N. Neovascularization in Achilles tendinopathy: have we been chasing a red herring? Knee Surg Sport Tra A. 2012; 20: 1891–4. 10.1007/s00167-012-2172-6 22890896

[56] Longo UG, Ramamurthy C, Denaro V, Maffulli N. Minimally invasive stripping for chronic Achilles tendinopathy. Disabil Rehabil. 2008; 30: 1709–13. doi: 10.1080/09638280701786922 18608390 10.1080/09638280701786922

[57] Korhonen RK, Laasanen MS, Toyras J, Rieppo J, Hirvonen J, Helminen HJ, et al Comparison of the equilibrium response of articular cartilage in unconfined compression, confined compression and indentation. J Biomech. 2002; 35: 903–9. 12052392 10.1016/s0021-9290(02)00052-0

[58] Lynch HA, Johannessen W, Wu JP, Jawa A, Elliott DM. Effect of fiber orientation and strain rate on the nonlinear uniaxial tensile material properties of tendon. J Biomech Eng. 2003; 125: 726–31. 14618932 10.1115/1.1614819

[59] Dakin SG, Werling D, Hibbert A, Abayasekara DRE, Young NJ, Smith RKW, et al Macrophage sub-populations and the lipoxin A4 receptor implicate active inflammation during equine tendon repair. Plos One. 2012; 7(2): e32333 doi: 10.1371/journal.pone.0032333 22384219 10.1371/journal.pone.0032333 PMC3284560

[60] Riley GP. Gene expression and matrix turnover in overused and damaged tendons. Scand J Med Sci Spor. 2005;15(4):241–51. 10.1111/j.1600-0838.2005.00456.x 15998341

[61] Watts AE, Nixon AJ, Yeager AE, Mohammed HO. A collagenase gel/physical defect model for controlled induction of superficial digital flexor tendonitis. Equine Vet J. 2012; 44: 576–86. doi: 10.1111/j.2042-3306.2011.00471.x 21950378 10.1111/j.2042-3306.2011.00471.x

[62] Lee KKH, Cai DQ, Tang MK, Tsang KF, Kwong WH, Chow PH. Growth arrest-specific 2 gene expression during patellar tendon healing. Cells Tissues Organs. 2003; 173:138–46. 12673096 10.1159/000069473

[63] Gelberman RH, Manske PR, Vande Berg JS, Lesker PA, Akeson WH. Flexor tendon repair in vitro: a comparative histologic study of the rabbit, chicken, dog, and monkey. J Orthopaed Res. 1984; 2: 39–48. 10.1002/jor.1100020107 6491797

[64] Kajikawa Y, Morihara T, Watanabe N, Sakamoto H, Matsuda K, Kobayashi M, et al GFP chimeric models exhibited a biphasic pattern of mesenchymal cell invasion in tendon healing. J Cell Physiol. 2007; 210: 684–91. doi: 10.1002/jcp.20876 17154365 10.1002/jcp.20876

[65] Lui PPY, Chan LS, Lee YW, Fu SC, Chan KM. Sustained expression of proteoglycans and collagen type III/type I ratio in a calcified tendinopathy model. Rheumatology. 2010; 49: 231–9. doi: 10.1093/rheumatology/kep384 19955224 10.1093/rheumatology/kep384

[66] Riley GP, Harrall RL, Constant CR, Chard MD, Cawston TE, Hazleman BL. Tendon degeneration and chronic shoulder pain–changes in the collagen composition of the human rotator cuff tendons in rotator cuff tendinitis. Ann Rheumatic Dis. 1994; 53: 359–66. 10.1136/ard.53.6.359 PMC1005350 8037494

[67] Lapiere CM, Nusgens B, Pierard GE. Interaction between collagen type-1 and type -3 in conditioning bundles organization. Connect Tissue Res. 1977; 5: 21–9. 141359 10.3109/03008207709152608

[68] Thornton GM, Shao X, Chung M, Sciore P, Boorman RS, Hart DA, et al Changes in mechanical loading lead to tendonspecific alterations in MMP and TIMP expression: influence of stress deprivation and intermittent cyclic hydrostatic compression on rat supraspinatus and Achilles tendons. Br J Sports Med. 2010; 44: 698–703. doi: 10.1136/bjsm.2008.050575 18801769 10.1136/bjsm.2008.050575

[69] Lo IKY, Marchuk LL, Hollinshead R, Hart DA, Frank CB. Matrix metalloproteinase and tissue inhibitor of matrix metalloproteinase mRNA levels are specifically altered in torn rotator cuff tendons. Am J Sports Med. 2004; 32:1223–9. doi: 10.1177/0363546503262200 15262646 10.1177/0363546503262200

[70] Gardner K, Lavagnino M, Egerbacher M, Arnoczky SP. Re-establishment of cytoskeletal tensional homeostasis in lax tendons occurs through an actin-mediated cellular contraction of the extracellular matrix. J Orthopaed Res. 2012; 30: 1695–701. 10.1002/jor.22131 22517354

[71] Lavagnino M, Arnoczky SP, Egerbacher M, Gardner KL, Burns ME. Isolated fibrillar damage in tendons stimulates local collagenase mRNA expression and protein synthesis. J Biomech. 2006; 39: 2355–62. doi: 10.1016/j.jbiomech.2005.08.008 16256123 10.1016/j.jbiomech.2005.08.008

[72] Knäuper V, López-Otin C, Smith B, Knight G, Murphy G. Biochemical characterization of human collagenase-3. J Biol Chem. 1996; 271: 1544–50. 8576151 10.1074/jbc.271.3.1544

[73] Le Graverand MPH, Eggerer J, Sciore P, Reno C, Vignon E, Otterness I, et al Matrix metalloproteinase-13 expression in rabbit knee joint connective tissues: influence of maturation and response to injury. Matrix Biol. 2000; 19: 431–41. 10980419 10.1016/s0945-053x(00)00093-7

[74] Brew K, Dinakarpandian D, Nagase H. Tissue inhibitors of metalloproteinases: evolution, structure and function. Biochim Biophys Acta. 2000; 1477: 267–83. 10708863 10.1016/s0167-4838(99)00279-4

[75] Anand-Apte B, Pepper MS, Voest E, Montesano R, Olsen B, Murphy G, et al Inhibition of angiogenesis by tissue inhibitor of metalloproteinase-3. Invest Ophth Vis Sci. 1997; 38: 817–23. 9112976

[76] Orchard J, Massey A, Brown R, Cardon-Dunbar A, Hofmann J. Successful management of tendinopathy with injections of the MMP-inhibitor aprotinin. Clin Orthop Relat R. 2008; 466: 1625–32. 10.1007/s11999-008-0254-z PMC2505252 18449616

[77] Arnoczky SP, Lavagnino M, Egerbacher M, Caballero O, Gardner K. Matrix metalloproteinase inhibitors prevent a decrease in the mechanical properties of stress-deprived tendons. Am J Sport Med. 2007; 35: 763–9. 10.1177/0363546506296043 17293464

[78] Ezura Y, Chakravarti S, Oldberg Å, Chervoneva I, Birk DE. Differential expression of lumican and fibromodulin regulate collagen fibrillogenesis in developing mouse tendons. J Cell Bio. 2000; 151: 779–88. 11076963 10.1083/jcb.151.4.779 PMC2169450

[79] Geng YQ, McQuillan D, Roughley PJ. SLRP interaction can protect collagen fibrils from cleavage by collagenases. Matrix Biol. 2006; 25:484–91. doi: 10.1016/j.matbio.2006.08.259 16979885 10.1016/j.matbio.2006.08.259

[80] Monfort J, Tardif G, Reboul P, Mineau F, Roughley P, Pelletier J-P, et al Degradation of small leucine-rich repeat proteoglycans by matrix metalloprotease-13: identification of a new biglycan cleavage site. Arthritis Res Ther. 2006; 8: R26–34. doi: 10.1186/ar1873 16507124 10.1186/ar1873 PMC1526547

[81] Kalamajski S, Oldberg A. The role of small leucine-rich proteoglycans in collagen fibrillogenesis. Matrix Biol. 2010; 29: 248–53. doi: 10.1016/j.matbio.2010.01.001 20080181 10.1016/j.matbio.2010.01.001

[82] Juneja SC, Veillette C. Defects in Tendon, Ligament, and Enthesis in Response to Genetic Alterations in Key Proteoglycans and Glycoproteins: A Review. Arthritis. 2013; article 154812 http://dx.doi.org/10.1155/2013/154812 10.1155/2013/154812 PMC3842050 24324885

[83] Zhang G, Ezura Y, Chervoneva I, Robinson PS, Beason DP, Carine ET, et al Decorin regulates assembly of collagen fibrils and acquisition of biomechanical properties during tendon development. J Cell Biochem. 2006; 98: 1436–49. doi: 10.1002/jcb.20776 16518859 10.1002/jcb.20776

[84] Nakamura N, Hart DA, Boorman RS, Kaneda Y, Shrive NG, Marchuk LL, et al Decorin antisense gene therapy improves functional healing of early rabbit ligament scar with enhanced collagen fibrillogenesis in vivo. J Orthopaed Res. 2000; 18: 517–23. 10.1002/jor.1100180402 11052486

[85] Hosaka Y, Kirisawa R, Mafune N, Takehana K. Downregulation of decorin and transforming growth factor-β1 by decorin gene suppression in tendinocytes. Connect Tissue Res. 2005; 46: 18–26. doi: 10.1080/03008200590935510 16019410 10.1080/03008200590935510

[86] Hosaka YZ, Uratsuji T, Ueda H, Uehara M, Takehana K. Comparative study of the properties of tendinocytes derived from three different sites in the equine superficial digital flexor tendon. Biomed Res. 2010; 31: 35–44. 20203418 10.2220/biomedres.31.35

[87] John T, Lodka D, Kohl B, Ertel W, Jammrath J, Conrad C, et al Effect of pro-inflammatory and immunoregulatory cytokines on human tenocytes. J Orthopaed Res. 2010; 28: 1071–7. 10.1002/jor.21079 20127972

[88] Dyson SJ. Medical management of superficial digital flexor tendonitis: a comparative study in 219 horses (1992–2000). Equine Vet J. 2004; 36: 415–9. 15253082 10.2746/0425164044868422

[89] Strom AC, Casillas MM. Achilles Tendon Rehabilitation. Foot Ankle. 2009; 14: 773–82. 10.1016/j.fcl.2009.08.003 19857848

[90] Gajhede-Knudsen M, Ekstrand J, Magnusson H, Maffulli N. Recurrence of Achilles tendon injuries in elite male football players is more common after early return to play: an 11-year follow-up of the UEFA Champions League injury study. Br J Sports Med. 2013; 47: 763–8. doi: 10.1136/bjsports-2013-092271 23770660 10.1136/bjsports-2013-092271

[91] Genovese R, Rantanen N, Simpson B. The use of ultrasonography in the diagnosis and management of injuries to the equine limb. Comp Cont Educ Vet. 1987; 9: 945–955.

[92] Genovese R, Reef V, Longo K, Byrd J, Davis W. Superficial digital flexor tendonitis: long term sonographic and clinical study of racehorses. In: Rantanen NW and Hauser ML, editors. Proceedings of the Dubai Equine International Symposium, pp 187–205; 1996.

[93] Andersson G, Forsgren S, Scott A, Gaida JE, Stjernfeldt JE, Lorentzon R, et al Tenocyte hypercellularity and vascular proliferation in a rabbit model of tendinopathy: contralateral effects suggest the involvement of central neuronal mechanisms. Br J Sports Med. 2011; 45: 399–406. doi: 10.1136/bjsm.2009.068122 20605910 10.1136/bjsm.2009.068122

[94] Alfredson H, Spang C, Forsgren S. Unilateral surgical treatment for patients with midportion Achilles tendinopathy may result in bilateral recovery. Br J Sports Med. 2014; 48: 1421–4. doi: 10.1136/bjsports-2012-091399 23193327 10.1136/bjsports-2012-091399

[95] Lui PPY, Maffulli N, Rolf C, Smith RKW. What are the validated animal models for tendinopathy? Scand J Med Sci Spor. 2011; 21: 3–17. 10.1111/j.1600-0838.2010.01164.x 20673247

[96] Oakes B. Tissue healing and repair: Tendons and ligaments In: Frontera WR, editor. Rehabilitation of sports injuries: scientific basis. Boston Massachusetts: Blackwell Science 2003 pp 56–99.

