# Supplementary material for: Spatiotemporal variations in gene expression, histology and biomechanics in an ovine model of tendinopathy
Source: PLoS One. 2017 Oct 12;12(10):e0185282. doi: 10.1371/journal.pone.0185282 (PMC5638251; doi:10.1371/journal.pone.0185282)
Supplement: S1 Table — Analysed genes (with standard abbreviations as used in the text), gene sequence accession numbers, primer sequences, annealing temperatures and product sizes are shown. (DOCX) [file pone.0185282.s001.docx]

| Target gene | Sequence Accession number | Sequence | Annealing temperature °C | Product size, bp |
| --- | --- | --- | --- | --- |
| Aggrecan (*ACAN)* | U76615 | F: TCA CCA TCC CCT GCT ACT TCA TC  R: TCT CCT TGG AAA TGC GGC TC | 58 | 105 |
| Versican (*VCAN*) | NM_181035 | F: CAT CTC ACC AGT ATC CTG TCT CAC G  R: AGT GTG CTG CCA TCA GTC CAA C | 55 | 128 |
| Decorin (*DCN*) | AF125041 | F: CCA AAG TGC GAA AGT CTG TGT TC  R: CAG CAA TGC GGA TGT AGG AGA G | 54 | 138 |
| Fibromodulin (*FMOD*) | X16485 | F: GCT CCA TCT TGA CCA CAA G  R: CCT TTC ATA GAA CTG CC ACT TCC | 55 | 123 |
| Lumican (*LUM*) | NM_173934 | F: TGG CTG ATA GTG GAG TTC CTG G  R: GGT TTT CAT TGA CTG TCG GTA TGC | 50 | 105 |
| Biglycan (*BGN*) | AF034842 | F: TGA TTG AGA ACG GGA GCC TGA G  R: TTT GGT GAT GTT GTT GGT GTG C | 56 | 143 |
| Collagen I (*COL1A1*) | AF129287 | F: ATC CCT GGA CAA CCT GGA CTT C  R: TCA TCA TAG CCG TAA GAC AAC TGG | 57 | 107 |
| Collagen II (*COL2A1*) | X02420 | F: TGA CCT GAC GCC CAT TCA TC  R: TTT CCT GTC TCT GCC TTG ACC C | 55 | 154 |
| Collagen III (*COL3A1*) | L47641 | F: GCA GGG AAC AAC TTG ATG GTG C  R: AAT AGT GGG ATG AAG CAG AGC G | 55 | 144 |
| *ADAMTS4* | NM_181667 | F: AAC TCG AAG CAA TGC ACT GGT  R: TGC CCG AAG CCA TTG TCT A | 60 | 149 |
| *ADAMTS5* | AF192771 | F: GCA TTG ACG CAT CCA AAC CC  R: CGT GGT AGG TCC AGC AAA CAG TTA C | 55 | 97 |
| *MMP2* | AF267159 | F: TGC TAC CAC CTC CAA CTA CGA TG  R: GTG CCA GTA TCA ATG TCA GGG G | 60 | 240 |
| *MMP9* | X78324 | F: AGG TGA ATC AGG TGG ACT ATG TGG  R: AGA AAG GAA GGT GGG AAG AGA GG | 59 | 221 |
| *MMP13* | AF267159 | F: CGT ATT GAT GCT GCC TAT G  R: GCT CCA GAC TTG GTT TTC T | 58 | 216 |
| *TIMP1* | S67450 | F: GGT TCA GTG CCT TGA GAG ATG C  R: GGG ATA GAT GAG CAG GGA AAC AC | 57 | 265 |
| *TIMP2* | M32303 | F: ACT CTG GCA ACG ACA TCT ACG G  R: TCT TCT TCT GGG TGG CAC TCA G | 57 | 261 |
| *TIMP3* | NM_174473 | F: CTT CCT TTG CCC TTC TCT ACC C  R: TCT GGT CAA CCC AAG CAT CG | 57 | 286 |
| *GAPDH* | U94889 | F: CCT GGA GAA ACC TGC CAA GTA TG  R: GT AGA AGA GTG AGT GTC GCT GTT G | 58 | 139 |
