# Supplementary material for: Spatiotemporal variations in gene expression, histology and biomechanics in an ovine model of tendinopathy
Source: PLoS One. 2017 Oct 12;12(10):e0185282. doi: 10.1371/journal.pone.0185282 (PMC5638251; doi:10.1371/journal.pone.0185282)
Supplement: S2 Table — Histology scoring parameters are shown. Slides were scored by three separate observers and a mean score calculated. The total histopathology score was calculated by adding all scores (except the proteoglycan score). A higher score indicated a tendon with a more pathological appearance. (DOCX) [file pone.0185282.s002.docx]

| **Outcome measure** | **Stain** | **Score** | **Description** |
| --- | --- | --- | --- |
| Cellularity | H&E | 0 | Normal |
|  |  | 1 | Mild increase |
|  |  | 2 | Moderate increase |
|  |  | 3 | Marked increase |
|  |  | 4 | Decrease |
| Tenocyte morphology | H&E | 0 | 75-100% tenocytes normal/spindle-shaped  (0-25% tenocytes abnormal/rounded) |
|  |  | 1 | 50-75% tenocytes normal/spindle-shaped  (25-50% tenocytes abnormal/rounded) |
|  |  | 2 | 25-50% tenocytes normal/spindle-shaped  (50-75% tenocytes abnormal/rounded) |
|  |  | 3 | 0-25% tenocytes normal/spindle-shaped  (75-100% tenocytes abnormal/rounded) |
| Vascularity | H&E | 0 | Normal |
|  |  | 1 | Mild increase |
|  |  | 2 | Moderate increase |
|  |  | 3 | Marked increase |
| Interfascicular infiltration | H&E | 0 | Normal |
|  |  | 1 | Mild increase |
|  |  | 2 | Moderate increase |
|  |  | 3 | Marked increase |
| Collagen fibre alignment | Picro-sirius red | 0 | 75-100% normal alignment |
|  |  | 1 | 50-75% normal alignment |
|  |  | 2 | 25-50% normal alignment |
|  |  | 3 | 0-25% normal alignment |
| Proteoglycan content | Toluidine blue | 0 | Normal |
|  |  | 1 | Mild increase |
|  |  | 2 | Moderate increase |
|  |  | 3 | Marked increase |
