# Supplementary material for: Spatiotemporal variations in gene expression, histology and biomechanics in an ovine model of tendinopathy
Source: PLoS One. 2017 Oct 12;12(10):e0185282. doi: 10.1371/journal.pone.0185282 (PMC5638251; doi:10.1371/journal.pone.0185282)
Supplement: S3 Table — Ratios are presented for non-operated controls (contralateral tendons and sham operated) and for both operated and non-operated tendons. CI = confidence intervals; 5% significance level is P<0.025 after Benjamini-Hochberg correction. (DOCX) [file pone.0185282.s003.docx]

| Score | Distal |  | Distance from cut |  | Time (weeks) |  | Surgery |  |
| --- | --- | --- | --- | --- | --- | --- | --- | --- |
| Variable | OR (95% CI) | *P* | OR (95% CI) | *P* | OR (95% CI) | *P* | OR (95% CI) | *P* |
| *Non-operated controls only* | |  |  |  |  |  |  |  |
| Sum of scores | 1.2 (0.5–2.6) | 0.44 | **0.23 (0.15–0.34)** | **<0.001** | 0.80 (0.64–1.00) | 0.054 | – |  |
| Cellularity | 1.2 (0.5–3.0) | 0.68 | **0.21 (0.13–0.34)** | **<0.001** | 0.85 (0.67–1.1) | 0.17 | – |  |
| Cell morphology | 1.1 (0.5–2.5) | 0.78 | **0.19 (0.12–0.30)** | **<0.001** | **0.60 (0.12–0.93)** | **0.009** | – |  |
| Vascularity | 1.5 (0.6–3.6) | 0.36 | **0.35 (0.23–0.52)** | **<0.001** | 0.91 (0.76–1.1) | 0.29 | – |  |
| Cell infiltration | 0.51 (0.17–1.6) | 0.24 | **0.20 (0.09–0.44)** | **<0.001** | 0.86 (0.67–1.1) | 0.21 | – |  |
| Alignment | 1.1 (0.4–2.8) | 0.84 | 1.1 (0.7–1.5) | 0.76 | 0.9 (0.7–1.2) | 0.72 | – |  |
| Proteoglycan | **4.8 (1.9-12)** | **0.001** | **0.24 (0.15–0.37)** | **<0.001** | 0.84 (0.65–1.1) | 0.20 | – |  |
|  |  |  |  |  |  |  |  |  |
| *ALL* |  |  |  |  |  |  |  |  |
| Sum of scores | 1.2 (0.7–2.1) | 0.54 | **0.17 (0.12–0.23)** | **<0.001** | **0.85 (0.74–0.97)** | **0.017** | **17 (8–38)** | **<0.001** |
| Cellularity | 1.0 (0.5–1.9) | 0.98 | **0.16 (0.11–0.23)** | **<0.001** | 0.91 (0.79–1.04) | 0.17 | **14 (7–28)** | **<0.001** |
| Cell morphology | 1.1 (0.6–2.0) | 0.80 | **0.16 (0.11–0.23)** | **<0.001** | **0.83 (0.72–0.96)** | **0.012** | **12 (6–23)** | **<0.001** |
| Vascularity | 1.2 (0.7–2.2) | 0.55 | **0.25 (0.18–0.34)** | **<0.001** | 0.91 (0.83–1.0) | 0.042 | **11 (6–23)** | **<0.001** |
| Cell infiltration | 0.65 (0.31–1.4) | 0.25 | **0.12 (0.07–0.20)** | **<0.001** | **0.80 (0.69–0.94)** | **0.005** | **58 (17–196)** | **<0.001** |
| Alignment | 1.5 (0.8–2.8) | 0.25 | **0.50 (0.38–0.66)** | **<0.001** | 0.93 (0.78–1.1) | 0.39 | **10 (5–21)** | **<0.001** |
| Proteoglycan | **2.2 (1.2–4.2)** | **0.015** | **0.21 (0.15–0.29)** | **<0.001** | 0.93 (0.80–1.1) | 0.40 | **2.6 (1.3–5.3)** | **0.007** |
